# Whole metagenome profiles of particulates collected from the International Space Station

**DOI:** 10.1186/s40168-017-0292-4

**Published:** 2017-07-17

**Authors:** Nicholas A. Be, Aram Avila-Herrera, Jonathan E. Allen, Nitin Singh, Aleksandra Checinska Sielaff, Crystal Jaing, Kasthuri Venkateswaran

**Affiliations:** 1 Physical and Life Sciences Directorate, Lawrence Livermore National Laboratory, Livermore, CA USA; 2 Computation Directorate, Lawrence Livermore National Laboratory, Livermore, CA USA; 3 Biotechnology and Planetary Protection Group, Jet Propulsion Laboratory, California Institute of Technology, M/S 89-2, 4800 Oak Grove Dr., Pasadena, CA 91109 USA; 4 Present Address: Department of Ecology, Evolution and Organismal Biology, Iowa State University, Ames, IA USA

**Keywords:** International Space Station, Microbiome, Functional metagenomics, Built environment, Cleanroom, Propidium monoazide

## Abstract

**Background:**

The built environment of the International Space Station (ISS) is a highly specialized space in terms of both physical characteristics and habitation requirements. It is unique with respect to conditions of microgravity, exposure to space radiation, and increased carbon dioxide concentrations. Additionally, astronauts inhabit a large proportion of this environment. The microbial composition of ISS particulates has been reported; however, its functional genomics, which are pertinent due to potential impact of its constituents on human health and operational mission success, are not yet characterized.

**Methods:**

This study examined the whole metagenome of ISS microbes at both species- and gene-level resolution. Air filter and dust samples from the ISS were analyzed and compared to samples collected in a terrestrial cleanroom environment. Furthermore, metagenome mining was carried out to characterize dominant, virulent, and novel microorganisms. The whole genome sequences of select cultivable strains isolated from these samples were extracted from the metagenome and compared.

**Results:**

Species-level composition in the ISS was found to be largely dominated by *Corynebacterium ihumii* GD7, with overall microbial diversity being lower in the ISS relative to the cleanroom samples. When examining detection of microbial genes relevant to human health such as antimicrobial resistance and virulence genes, it was found that a larger number of relevant gene categories were observed in the ISS relative to the cleanroom. Strain-level cross-sample comparisons were made for *Corynebacterium*, *Bacillus*, and *Aspergillus* showing possible distinctions in the dominant strain between samples.

**Conclusion:**

Species-level analyses demonstrated distinct differences between the ISS and cleanroom samples, indicating that the cleanroom population is not necessarily reflective of space habitation environments. The overall population of viable microorganisms and the functional diversity inherent to this unique closed environment are of critical interest with respect to future space habitation. Observations and studies such as these will be important to evaluating the conditions required for long-term health of human occupants in such environments.

**Electronic supplementary material:**

The online version of this article (doi:10.1186/s40168-017-0292-4) contains supplementary material, which is available to authorized users.

## Background

The microbial content of built environments is an area of increasing study, particularly with the expansion of culture-independent sequence-based assessments [1, 2]. The ecology of indoor environments with continuous human contact is of great interest due to potential impact on human health; this is of particular concern in built environments that are spatially confined with long-term human occupants [3, 4]. These environments are typified by enclosures associated with extraterrestrial habitation. The National Aeronautics and Space Administration (NASA) has a strong interest and motivation to understand the microbial content and ecology of these environments, particularly the International Space Station (ISS) as a test bed for other analogs of closed systems [5] and future human habitation on Mars [6].

Previous microbial assessments of the ISS have largely been restricted to the examination of smaller subsets of microorganisms using culture-based microbiology or quantitative PCR [7, 8]. Microbial survey units based on detection of specific bacterial biomarkers have also been deployed to the ISS [9]. Further studies have used Sanger sequencing in an effort to identify a panel of potential pathogens in potable water [10]. Recent examination of microbial content associated with astronauts has explored the degree to which the human microbiome adjusts to habitation within the ISS [11, 12]. Implications of these data for maintenance of crew health are critical when evaluating design and maintenance of this highly specialized built environment [6].

Previous studies at the Jet Propulsion Laboratory (JPL) and others expanded on this knowledge base by applying bacterial 16S ribosomal RNA (rRNA) and fungal internal transcribed spacer (ITS) amplicon sequencing for examination of broader microbial communities [13–15]. These studies were coupled with propidium monoazide (PMA) treatment, eliminating detection of non-cellular DNA and DNA associated with cells exhibiting a compromised membrane, and providing culture-independent quantification of viable microorganisms. These studies revealed important distinctions in diversity between the built environments of the ISS and terrestrial cleanrooms from the Spacecraft Assembly Facility (SAF) at JPL, demonstrating that the ISS microbiome is strongly impacted by human skin-associated microbes.

Through application of Illumina and pyrosequencing techniques to targeted amplicons, these previous studies provided a broader survey of the bacterial and fungal microbiome [13–16]. An inherent limitation of amplicon sequencing, however, is that the wider metagenome is not examined, which limits taxonomic resolution and prohibits a study of the functional genetic content of the microbiome. Knowledge of the functional capabilities of the microbiome could be critical to determining whether the population poses a potential threat to human health. Recent studies have just begun to explore the potential of whole metagenome data for examining the functional genomics of microbial communities associated with spacecraft in a terrestrial environment [17]. Further application of these techniques to space habitats will be critical. It has been suggested that the immune profile of astronauts may be modulated following exposure to microgravity and space travel [12, 18]; thus, the response to microbial presence and activity may be difficult to predict, and a complete portrait of that activity is important to risk assessment.

Of particular interest is the genetic potential for resistance to antimicrobials, as the presence of such microorganisms could significantly jeopardize health both during and after completion of the mission. To more thoroughly assess these factors, a whole metagenome sequencing approach to analyze both the ISS and SAF environments was undertaken. Samples from high-efficiency particulate arrestance (HEPA) filters and dust from within the ISS cabin as well as dust from the JPL-SAF were analyzed. As in the previous JPL study [14], samples were either processed directly or pre-treated with PMA to assess intact, presumably viable microbes, and resultant sequence data were examined for population abundance, functional genomic characterization, and phylogenetic relationships. Furthermore, metagenome mining was conducted to characterize dominant, virulent, and novel microorganisms by comparing the whole genome sequences of select cultivable strains from these samples.

## Methods

### Sample characteristics

Materials collected from three sample types examined as part of this study included the following: ISS filter particles obtained from HEPA systems onboard the space station (ISS filter), ISS dust samples obtained through vacuum collection within the cabin (ISS dust), and dust samples obtained through vacuum collection from a cleanroom where spacecraft are assembled (SAF dust). Portions of the cargo destined for the ISS are prepared in cleanrooms (class 100K) such as the SAF, where spacecraft are assembled; thus, any pre-existing commonality between the environments, derived from this subset of components, was of interest. Due to low biomass limitations in the availability of these materials, one sample was analyzed for each group. However, about 1 g of particulate materials was aseptically scraped from the HEPA filter and from the vacuum cleaner bags. These samples were either untreated to examine total microbial burden or subjected to PMA treatment to examine viable microbial content [14].

The sample characteristics, usage time of the material collection devices or system(s), model, make, and cleanroom conditions where the devices were used have been published elsewhere [14] but are briefly stated below. The materials collected using the HEPA filter system (40 months old) are representative of circulating air, and the vacuum cleaner bag was representative of fixed ISS (1-day collection) or JPL-SAF (180 days) surfaces. Reagent and water controls for DNA extraction, PCR, and no-template negative controls were also included in this study. Molecular microbial community analyses, based on amplification of bacterial 16S regions and fungal ITS regions, have been previously documented for the ISS filter, ISS dust, and SAF dust [15]. The previous iTag-based (Illumina) molecular characterization was extended in this study by applying whole metagenome sequencing using the same archived DNA aliquots [14, 15]. Results from the previous and current study are discussed here for comparative purposes.

### Sample processing

Vacuumed dust samples were collected and weighed, whereas the HEPA filter elements were divided into small pieces and particulates associated with the pieces were aseptically collected using sterile scalpels before being quantitatively measured. Approximately 1 g of each vacuum dust and HEPA filter-associated particles was weighed, placed into a sterile tube containing 25 mL of sterile phosphate-buffered saline (PBS), and vortexed for 1 min. After vigorous mixing, large particles were allowed to settle, and aliquots of samples were carefully siphoned and DNA extracted.

### Sample processing for molecular analysis

The biological materials associated with each sample (15 mL) were further concentrated using Amicon Ultra-50 Ultracel centrifugal filter tubes (Millipore). Each filter unit has a molecular mass cutoff of 50 kDa, which facilitates the concentration of microbial cells, spores, and exogenous nucleic acid fragments greater than 100 bp in a final volume of 2.5 mL. All filtered samples were then divided into three separate aliquots: the first aliquot (1000 μL) was subjected to PMA pre-treatment (viability assessment), the second (1000 μL) was an untreated environmental sample (viable + nonviable; i.e., total DNA), and the third (500 μL) was archived for other molecular characterizations [14].

For measuring the viable microbial population, one aliquot of filter-concentrated sample suspension (1000 μL) was treated with 12.5 μL of PMA (2 mM; Biotium, Inc.) to a final concentration of 25 μM [19, 20], followed by thorough mixing and incubation in the dark for 5 min at room temperature [21]. The sample was exposed to the PhAST Blue-PhotoActivation System (GenIUL, S.L.) for 15 min (in parallel with the non-PMA-treated sample). This step facilitates the blocking of DNA from dead cells [21]. The samples were then split in half; one half was subjected to bead beating with the Fastprep-24 bead-beating instrument (MP Biomedicals) with parameters set at 5 m/s for 60 s. The second half of the unprocessed sample was combined with the mechanically disrupted counterpart before DNA was extracted via the Maxwell 16 automated system (Promega), in accordance with the manufacturer’s instructions [22]. Resulting DNA suspensions (100 μL each) were stored at −20 °C.

### Whole genome amplification

Extremely limited quantities of biomass were available for study, with all extracted DNA samples yielding <1 ng/μL; thus, a whole genome amplification step was necessary. All nucleic acid samples were subjected to multiple displacement amplification (MDA) using the phi29-based Repli-g system (Qiagen). This platform has been shown to impart less amplification-based bias when compared to comparable systems [23, 24]. Amplified samples were purified using the DNA Clean and Concentrator Kit (Zymo Research).

### Library preparation and sequencing

DNA libraries were prepared for sequencing using the Nextera DNA Library Preparation Kit (Illumina). Quality and fragment size were assessed on the Bioanalyzer 2100 (Agilent). Libraries were normalized to 2 nM, pooled, denatured, and diluted to 1.8 pM according to the manufacturer’s standard recommendations (Illumina). Sequencing was performed on the NextSeq 500 with the NextSeq Series High Output Kit v2 (Illumina), using 150-bp, paired-end reads. For the ISS dust, 37,297,848 and 36,062,308 raw reads were obtained for untreated and PMA-treated samples, respectively. For the ISS filter, raw read counts were 98,960,056 and 25,212,186, respectively. For the SAF dust, 57,301,138 and 38,946,886 raw reads were obtained, respectively.

### Sequence analysis

**Fig. 1:**
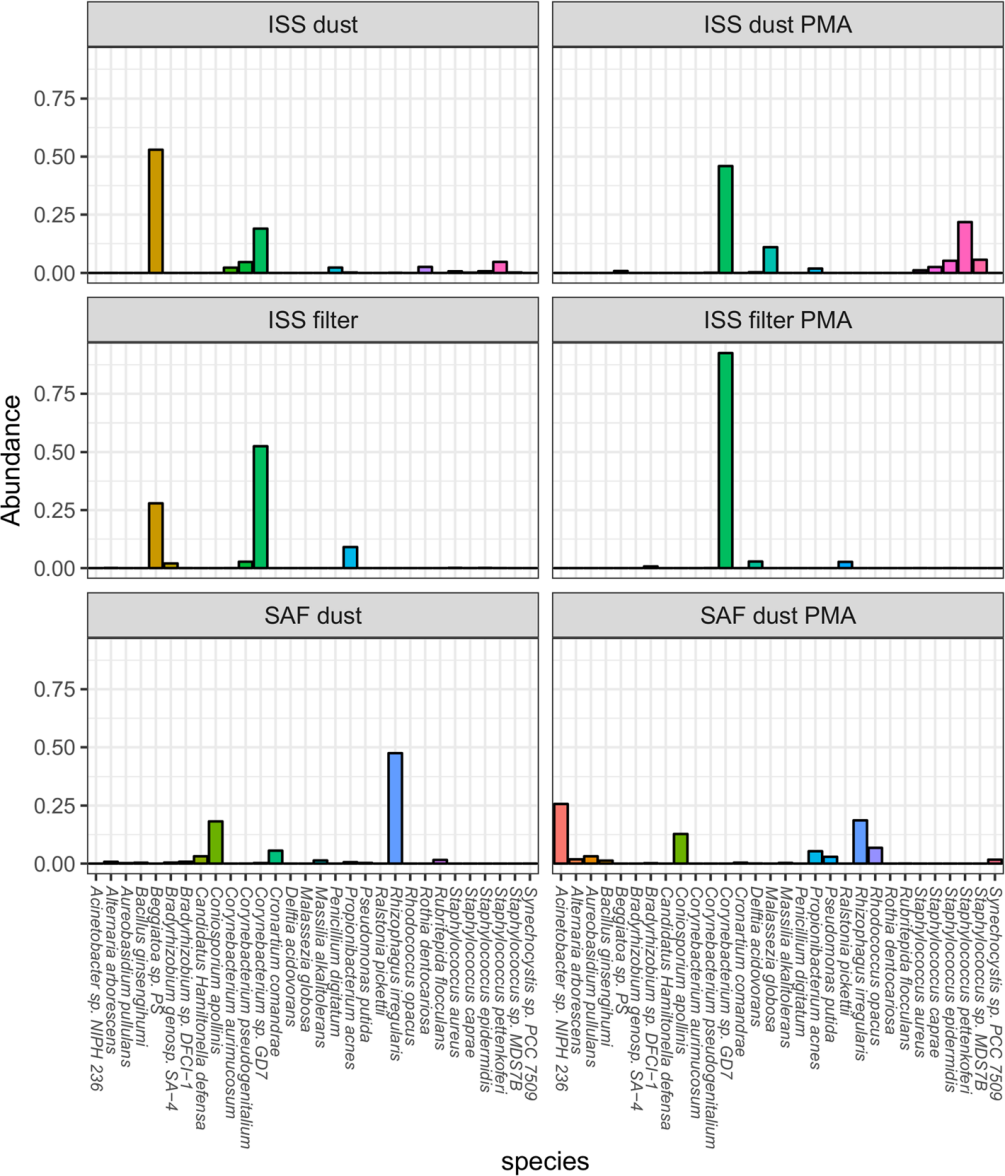
Species-level microbial composition of ISS and SAF samples. Sequence reads obtained from the ISS and SAF samples were uniquely mapped to microorganisms at species-level resolution. Relative abundance of the top 30 microbial species observed in total and viable (PMA-treated) populations was determined. The proportion of total mapped reads attributed to each of these top species is shown. Comparison of the microbial profile represented by the top 30 microbial species observed in each total and viable (PMA-treated) sample is shown. *Each panel* represents one independent sample. The top detected species across all samples are shown on the *horizontal axis* and relative read abundance along the *vertical axis*

**Fig. 2:**
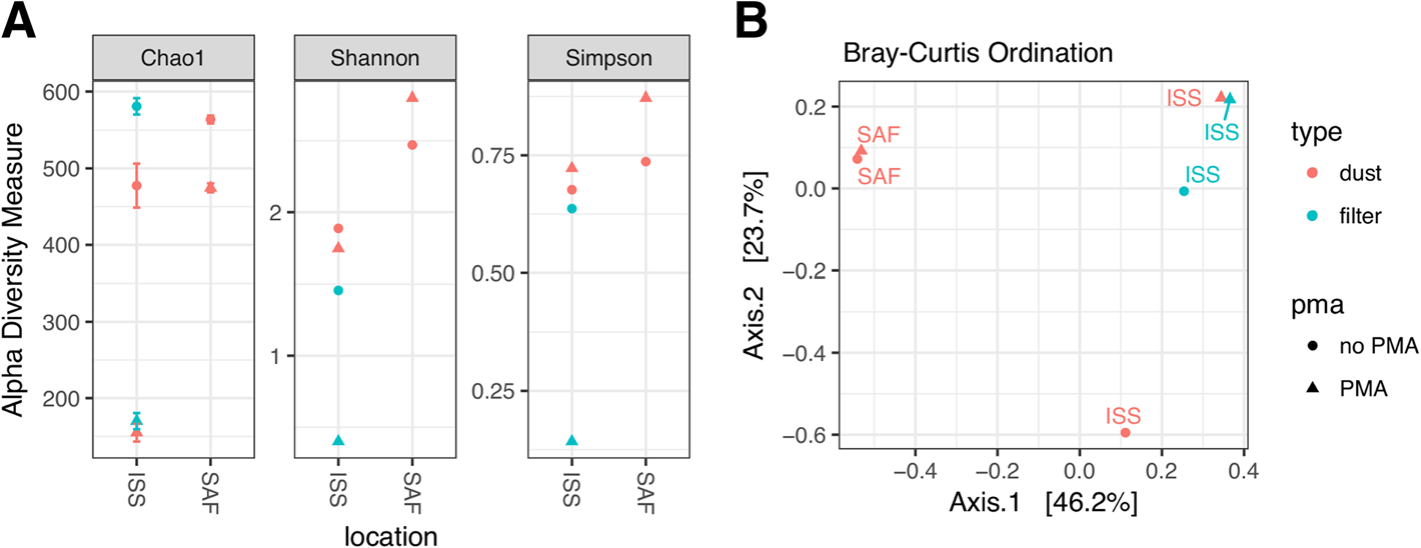
Diversity and ordination of ISS and SAF samples based on species-level microbial populations. Abundance of each microbial species, as determined by unique mapping of sequence reads obtained from each ISS and SAF sample, was used to perform ecological diversity analysis and sample-level ordination. **a** Diversity estimates for each sample type, as quantified by calculation of Chao1 richness and alpha diversity using the Shannon entropy and Gini-Simpson indices, based on absolute read counts. **b** Principal coordinate analysis using the Bray-Curtis distance of the ISS and SAF-derived samples based on absolute read counts. PMA-treated samples are shown in *triangles*, and untreated samples are shown in *dots*. Dust samples are shown in *orange color*, and filter samples are shown in *green color*

**Fig. 3:**
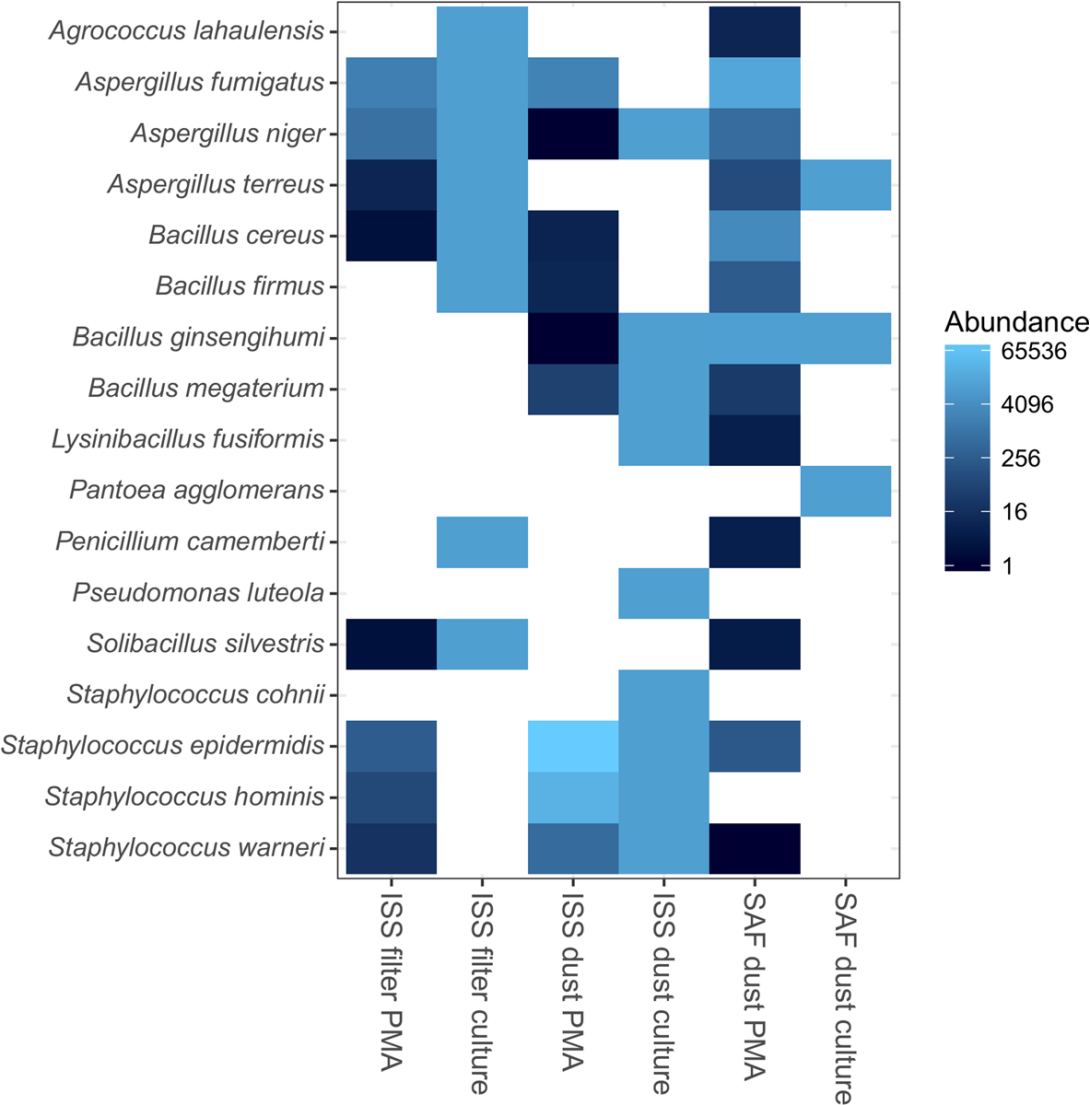
Metagenomic sequence data for microorganisms previously cultured from the ISS and SAF samples. Unfiltered, absolute read counts are shown for isolated microorganisms. Cultured microbes not present in the LMAT database were omitted. Each sample type (ISS filter, ISS dust, and SAF dust) is shown along the *horizontal axis*. For each sample type, results are shown for PMA-treated DNA and culture results. Culture status is shown as binary: positive (*bright blue*) or negative (*white*)

Sequence data were processed with the Livermore Metagenomics Analysis Toolkit (LMAT) (version 1.2.6) [25] using default settings. The relative quantity of uniquely mapped, species-specific paired reads corresponding to each taxonomic target was identified. A minimum read match score of 0.5 was applied to maintain high-confidence assignments in the taxonomic composition analysis (Figs. 1 and 2; Additional file 1: Figures S1 and S2). This minimum match score was not applied in the analysis comparing sequence detection to culture isolate data (Fig. 3) to facilitate identification of microorganisms which were present at low abundance but amenable to culture. Reads mapping to *Homo sapiens* were omitted from analysis. These reads represented 53 to 85% of species-specific sequence content in the ISS filter, 64 to 65% in the ISS dust, and 35 to 53% in the SAF dust.

For identification of individual microbial genes, read pairs were mapped using an identity cutoff threshold of 90%. A query read is greedily assigned to the gene with the highest fraction of matching 20-mers requiring at least 90% of the 20-mers to match. The threshold serves as a strict filtering criterion to focus on nearly identical matches. No minimum threshold for the length of a reference gene sequence recovered is required; thus, only partial genes may be recovered in some cases. Detected genes were screened for antimicrobial resistance using the Comprehensive Antimicrobial Resistance Database (CARD) [26]. Genes were screened for virulence factors by screening against the Virulence Factors Database (VFDB) [27]. For each gene database, the genes are stored as constituent 20-mers for sequence matching.

### Statistical analysis of microbiome data

The phyloseq package (version 1.14.0) in R was used for principal component analysis ordination and calculation of alpha diversity statistics, including the Chao1 richness estimate, Shannon entropy, and Gini-Simpson ecological indices [28]. Comparison of differential abundance between ISS and SAF environments, using a negative binomial generalized linear model, was performed using the DESeq2 package [29]. Each species’ log fold change between environments was evaluated with a Wald test. Results were filtered per defaults to optimize the number of species with adjusted *P* values below a false discovery rate of 10%. *P* values were adjusted for false discovery using the Benjamini and Hochberg (BH) correction. Permutational multivariate analysis of variance (PERMANOVA) analysis of Bray-Curtis distances was performed using the vegan package [30]. *P* values for marginal effect sizes were adjusted for testing multiple covariates (Location, Type, PMA-treatment) as above, using the BH correction. The presence of functional gene categories was identified by mapping sequence reads from all samples to individual microbial genes using LMAT as described above, followed by assignment to KEGG pathways. Unclassified reads were discarded. Visualization of functional pathway analysis was performed in MEGAN5 [31].

LMAT was used for the majority of sequence analysis due to its ability to perform robust taxa and functional read score filtering, in addition to onboard screening for contamination in reference sequences. However, the alternate methods below were used for taxonomic network analyses (Additional file 1: Figures S3, S4, S5 and S6) to facilitate incorporation into MEGAN5-based network tools. Alignments were carried out using DIAMOND [32] as described in the MEGAN5 manual. BIOM files generated with MEGAN5 were used for the development of a node table using QIIME (version 1.9.1) [33]. Cytoscape version 3.4 was used to visualize the node table [34].

### Mapping and variant calling

A schematic workflow outlining the variant analyses adapted during this study is given in Additional file 1: Figure S7. Short read pairs (median combined length 302 bp) were aligned, classified at the genus level by LMAT to chosen reference genomes, and called variants using the framework provided by Snippy (version 3.1) [35] as previously referenced [36]. Snippy conveniently wraps “bwa mem” [37] for alignment and “freebayes” [38] for variant calling. Snippy was run with default parameters “--mincov 10” (minimum depth), “--minfrac 0.9” (minimum non-reference allele fraction, not necessary in our analysis but included for completeness), and additionally, “--ungapped” to preserve unmapped reads for supplementary analysis.

We analyzed bases at reference positions that met the depth threshold of ten or more reads, and only considered substitutions (indels and complex variants were ignored). Furthermore, variants were decomposed to allelic primitives (gaps and mismatches of length 1 bp) using “vcfallelicprimitives” from the software library “vcflib” [39]. Identified variants were intersected with coding sequence coordinates using annotation provided by NCBI (gff accompanying reference genome assembly).

### Allele frequencies

Allele frequencies within each sample were directly estimated using the observed read counts supporting present alleles at each position (i.e., AO and RO fields in the vcf file generated by “freebayes”). The threshold for determining allele presence is three or more reads and 10% or more of read depth supporting the allele as previously referenced [40]. For variants in *Corynebacterium ihumii* GD7 regions that were mapped in all ISS samples, we plotted the relative abundance of each non-reference allele in each sample, sorted by the number of samples in which they were present, as well as the total fraction of reads.

### Consensus sequences

Samples using consensus sequences over *nearly fixed sites*, i.e., reference positions where the major allele constituted 90% or more of the read depth, were compared. Polymorphic loci and unmapped reference sequence were masked.

### Reference genomes

Given that pathogenic *Aspergillus* [36] and *Bacillus* [14] species were previously isolated and may exhibit enhanced virulence, the whole genome sequences of these selected species [41] were compared with the metagenomic sequences generated during this study [36]. *Corynebacterium* were also selected for similar analysis due to their dominance in ISS samples [14]. Genomic sequences and annotation were downloaded from NCBI (ftp://ftp.ncbi.nlm.nih.gov/genomes/). For *Corynebacterium*, reads were mapped to strain GD7 (accession: GCF_000403725.1_GD7), recently annotated as *C. ihumii* GD7 [42]. For *Aspergillus* and *Bacillus*, reads were pooled by location (ISS, SAF) and mapped to multiple representative reference genomes. *Aspergillus* reads were mapped to *Aspergillus fumigatus* strains Af293, CEA10/A1163, IF1SW-F4, and ISSFT-021 (accessions: GCF_000002655.1_ASM265v1, GCA_000150145.1_ASM15014v1, GCA_001643655.1_ASM164365v1, GCA_001643665.1_ASM164366v1), and *Bacillus* reads were mapped to *Bacillus cereus*, *Bacillus anthracis*, and *Bacillus thuringiensis* genomes (accessions: GCF_000007825.1_ASM782v1, GCF_000007845.1_ASM784v1, GCF_000008165.1_ASM816v1, GCF_000008505.1_ASM850v1, GCF_000497525.1_ASM49752v2).

Additionally, *Bacillus* reads were mapped to assemblies of four *B. cereus* sensu lato isolates previously recovered from the ISS. Assemblies were downloaded from ftp://ftp.cbcb.umd.edu/pub/data/issensis/asms/. The sequencing data for the assemblies of ISS *Bacillus* species are available from the NASA GeneLab system (accession: GLDS-64; https://genelab-data.ndc.nasa.gov/genelab/accession/GLDS-64/#).

## Results

### Metagenome-based microbial diversity

Relative abundance of each species was measured by assessing the relative number of species-specific reads corresponding to queried reference sequences. The majority of species-specific mapped sequence data could be attributed to a relatively small number of individual species (Fig. 1 and Additional file 1: Figure S1). Overall, 80.9 to 98.8% of mapped microbial sequence data was attributable to the 30 highest abundant species across all samples, as measured by summed relative abundance. The top 100 detected species represented 92.5 to 99.6% of total sequence data and comprised 65 total genera (Additional file 1: Figure S2). Pre-processing of samples with PMA allowed for selective detection of DNA derived from intact cells, providing a view of sequence data corresponding to viable microorganisms (Fig. 1). As viable microorganisms are most likely to be physiologically relevant to the microbiome of human-inhabited spaces, the following examination of microbial composition for each respective location is restricted to PMA-treated samples. In some cases, PMA-treated and untreated samples were included.

#### ISS filter

The PMA-treated ISS filter material was largely dominated by *Corynebacterium* (Fig. 1 and Additional file 1: Figure S1). In total, 25 published species of *Corynebacterium* were identified, including *Corynebacterium diphtheriae*, the causative agent of diphtheria. Additional human health-relevant members of the genus *Corynebacterium* retrieved included *Corynebacterium aurimucosum*, *Corynebacterium jeikeium*, *Corynebacterium pseudogenitalium*, and *Corynebacterium urealyticum*. The largest proportion of *Corynebacterium* sequence data corresponded to the non-pathogenic *C. ihumii* GD7. *Aspergillus* were also represented within fungal sequence data from the ISS filter. A total of four *Aspergillus* species were identified in the PMA-treated ISS filter sample, including *Aspergillus kawachii*, *Aspergillus nidulans*, *Aspergillus niger*, and *Aspergillus sojae*.

#### ISS dust

The viable population of ISS dust featured *Staphylococcus*, *Corynebacterium*, and *Propionibacterium* (Additional file 1: Figure S2). Detected *Staphylococcus* species included the human skin-associated *Staphylococcus aureus*, *Staphylococcus caprae*, *Staphylococcus pettenkoferi*, and *Staphylococcus epidermidis*. Reads corresponding to *Staphylococcus* phage were correspondingly detected in this sample. *C. ihumii* GD7 were observed at higher levels in the ISS samples relative to the SAF samples, though this difference was significant at a relaxed threshold of adj. *P* < 0.1 (*P* = 0.06) (Fig. 1). Fungal composition included a range of *Penicillium* species (Fig. 1 and Additional file 1: Figure S1), including *Penicillium aurantiogriseum*, *Penicillium chrysogenum*, *Penicillium digitatum*, *Penicillium nalgiovense*, and *Penicillium roqueforti*. The human scalp-associated fungus, *Malassezia globosa*, representing over 10% of species-specific microbial data in the PMA-treated ISS dust (Fig. 1), was also identified at higher levels in the ISS relative to SAF samples (*P* = 0.04).

#### SAF dust

SAF dust was compositionally distinct from ISS samples (Fig. 1 and Additional file 1: Figure S1). As noted above, SAF samples were lower in abundance of *C. ihumii* GD7 than in ISS samples, the PMA-treated SAF sample exhibiting the lowest relative abundance among all three PMA-treated samples. *Staphylococcus* species were similarly reduced relative to the ISS-derived samples, including *S. aureus* (*P* = 0.02), *S. epidermidis* (*P* = 0.006), and *S. pettenkoferi* (*P* = 3E-4). The bacterial population was largely composed of *Acinetobacter* sp. NIPH 236, *Propionibacterium acnes*, *Pseudomonas putida*, and *Rhodococcus opacus*. The relative abundance of several top fungal species was higher in SAF dust relative to ISS samples. Among the fungi observed at significantly higher levels in the SAF were the soil microorganism *Aureobasidium pullulans* (*P* = 0.006) (Fig. 1), the potentially pathogenic black yeast *Coniosporium apollinis* (*P* = 0.05), and the plant and opportunistic pathogen *Alternaria arborescens* (*P* = 0.006) which were also present at elevated levels in the SAF compared to the ISS samples. As was the case in the ISS filter, several *Aspergillus* species were observed, including *A. kawachii*, *A. niger*, and *A. fumigatus*.

### Alpha diversity and ordination analyses

Alpha diversity and richness estimates were calculated from absolute read counts without rarefying to an even depth so as to minimize data loss and include low abundant species detection [43] (Fig. 2a). It is anticipated that the cutoffs applied by LMAT will reduce noise within the data so as to minimize impact of library size-dependent sequence noise on calculated diversity metrics. Both the assessed dust samples obtained from the ISS and those obtained from the SAF trended toward greater microbial diversity than filter samples; however, our study is not designed to test for differences in diversity between many possible factors; thus, significance cannot be confidently assigned. The Chao1 estimator, Shannon entropy, and Simpson index highlight different aspects of the species diversity in the samples. The Chao1 richness estimate shows that PMA-treated samples (triangles) are trending toward a lower expected number of species, as might be expected when examining the smaller subset of viable microorganisms. However, within the two SAF samples, the Shannon and Simpson indices suggest that PMA treatment resulted in higher species evenness, despite the lower richness estimate. It is possible that there is a small number of non-viable species composing the majority of sequence data in untreated samples, saturating available detectable sequence. Removal of these species by PMA treatment may have allowed for the detection of a broader range of viable microorganisms, increasing observed diversity. This observation could also be due to novel species having attributable sequence reads split between multiple near-neighbor references, resulting in an amplified observed diversity metric. While these hypotheses are offered as possible explanations, it needs to be noted that feasibility limitations in sample size make it difficult to perform a confident assessment of relative richness/diversity.

Principal coordinate analysis of the samples was performed using Bray-Curtis distances based on raw read accounts of all microbes. ISS filter and dust microbial populations were observed to demonstrate a visually distinct profile from the SAF dust within the ordination space (Fig. 2b). PERMANOVA analysis revealed that this difference was not highly significant (*P* = 0.18), though given the drastically different environments, we expect further sampling would likely show this difference to be significant. No significant differences were observed in the distance between samples when grouped according to treatment status (untreated vs. PMA) or type (dust vs. filter).

### Taxonomic network analysis

A network analysis of all PMA-treated samples was performed to examine in more detail taxonomic commonality across samples (Additional file 1: Figure S3). This analysis illustrates, as a network, how each taxonomic class was distributed across PMA-treated samples, providing a visual representation of which taxa were observed as shared versus unique to a given sample type. The taxa used to perform this analysis were identified using an alternative sequence mapping approach (DIAMOND, as described in the “Methods”), as opposed to LMAT, to facilitate incorporation within the MEGAN5 pipeline. The genus-level alignment results from this approach are shown in Additional file 1: Figures S4-S6 and are comparable to those observed via LMAT. Taxa nodes shown as the same color as their corresponding sample node were unique to that sample, while gray taxa nodes were shared between two or three sample types. ISS dust and ISS filter samples shared the highest number of taxa at the class level, containing only one and five exclusively unique taxonomic classes, respectively. As was observed above, SAF dust composition was distinct from each of the ISS samples, exhibiting 54 unique classes not shared by the other two samples.

### Sequence detection of cultivated microorganisms from metagenomic dataset

The samples examined in this study were previously subjected to microbiological culture analysis [14]. The metagenomic data were mined to explore the presence of genetic signatures relevant to culture isolates from these samples, and the absolute numbers of unfiltered reads corresponding to the genomes of each isolated microorganism are depicted (Fig. 3). Unfiltered reads were analyzed to maximize the detection of low abundance microorganisms. The LMAT reference database contained reference genomes for 17 of the 31 cultured isolates identified at the species level. Species not present in the LMAT reference database were not included in this analysis. It is likely that sequence reads corresponding to isolates not present in the database were assigned to near-neighbor species. In total, 12 bacterial and five fungal species present in the LMAT database were isolated using conventional cultivation methods. Among these cultivable microorganisms, eight, nine, and three species were cultivated from the ISS filter, ISS dust, and SAF dust samples, respectively. *A. niger* was the only fungal species cultured from both ISS samples and was also detected in both sequence datasets. All other cultivable species were present in at least one of the samples tested. However, sequences of *A. fumigatus*, *B. cereus*, *S. epidermidis*, and *Staphylococcus warneri* were retrieved from PMA-treated (viable) portions of all three tested samples. There are two instances where cultured species were detected in total DNA, but not in PMA-treated samples (data not shown). Both of these taxa, *Pantoea agglomerans* and *Lysinibacillus fusiformis*, were present at extremely low abundance in PMA-untreated samples (between one and six reads).

### Functional pathway analysis

**Fig. 4:**
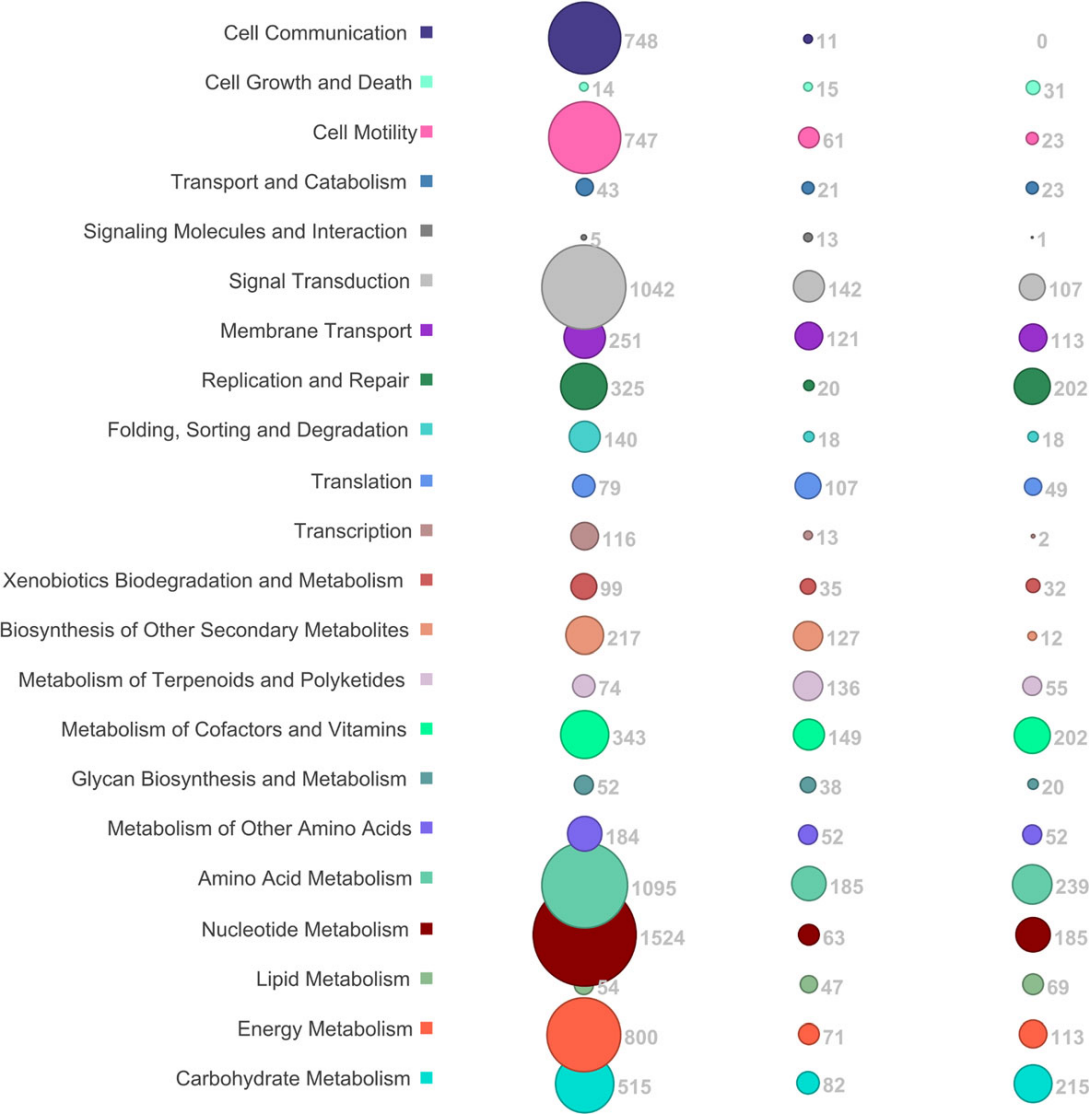
Microbial gene pathways observed in the whole metagenomes of ISS and SAF samples. Reads matching microbial gene targets above an identity threshold of 0.9 were assigned to KEGG orthologies. KO number was used to assign a gene function category, shown along the *vertical axis*. Read abundance is graphically represented on a square-root scale. Absolute read counts are shown adjacent to each corresponding *circle*

An inherent advantage of the applied whole metagenome approach is the ability to examine gene content of the microbial population. To examine the presence of a given gene, sequence reads from all samples were mapped to individual microbial genes, which were then assigned to KEGG pathways (Fig. 4). The microbial population within the ISS dust sample in particular exhibited enrichment over other samples for pathways associated with general microbial propagation, including nucleotide and amino acid metabolism, signal transduction, and cell motility and communication.

### Antimicrobial resistance profiles

**Fig. 5:**
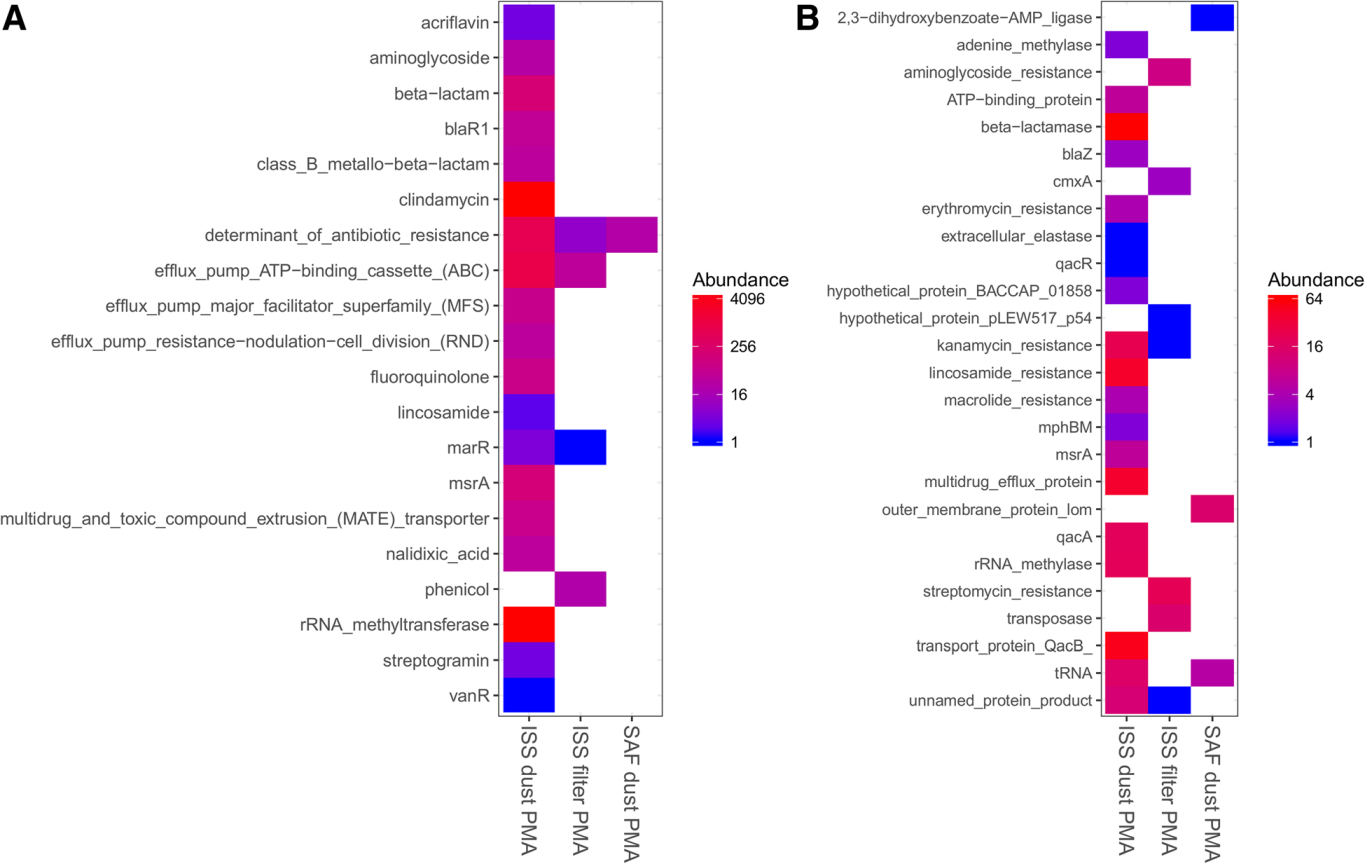
Resistance and virulence gene categories detected in the viable (PMA-treated) ISS and SAF samples. Genes uniquely identified by LMAT were screened against the Comprehensive Antimicrobial Resistance Database (CARD) and Virulence Factors Database (VFDB). Detected genes were binned into functional categories. Categories shown are **a** antimicrobial resistance and **b** virulence. The *color scales* indicate absolute read abundance. Gene categories are shown alphabetically along the *vertical axis* and PMA-treated samples along the *horizontal axis*

Microbial genes identified by LMAT were screened for antimicrobial resistance (AMR) factors using the CARD. Detected AMR genes were subsequently sorted into categories (Fig. 5a). Virtually no AMR signatures were detected within the SAF dust sample, in either the viable (PMA-treated) or total (Additional file 1: Figure S8) population. Within ISS samples, however, a range of AMR categories were identified, including resistance to aminoglycosides, beta-lactams, clindamycin, fluoroquinolones, lincosamide, streptomycin, and tetracycline. A larger proportion of AMR-associated sequence was observed in the ISS dust relative to filter samples. Significantly fewer AMR gene categories were observed in the PMA-treated ISS filter compared to the untreated ISS filter sample (*P* = 0.008, Fisher’s exact test).

### Virulence factor analysis

In addition to AMR analysis, microbial genes identified by LMAT were screened for virulence factors using the VFDB. Sequence reads corresponding to virulence genes were binned into functional categories to combine genes contributing to similar mechanisms of virulence (Fig. 5b). These include efflux proteins, transposases, methylases, and resistance to a range of antibiotics. There is substantial overlap between genes annotated as conferring AMR and those implicated in virulence; thus, a proportion of those shown are resistance associated. The identified AMR gene sets are not identical, however, due to distinctions between the CARD and VFDB databases.

A much lower quantity of sequence data was associated with virulence compared to AMR; however, a similar pattern of distribution was observed, in that the ISS dust was associated with the largest number of virulence categories and SAF dust the fewest. When compared with PMA-untreated samples, PMA treatment only resulted in a significant change in the number of detected virulence categories in the ISS filter (Additional file 1: Figure S9; *P* = 0.003, Fisher’s exact test). Despite the low total read count, reads are likely to be highly informative due to the strict filtering criteria applied. This provides confidence in the presence of a given virulence factor but does not indicate that our inability to detect any given virulence category substantiates its absence in a given sample.

### Metagenome sequence mining with LMAT single genomes

Since iTag sequence-based analyses (resolving only to the genus level) revealed the presence of *Corynebacterium* as the dominant bacterial genus [14], the metagenome sequences generated from these samples were mined to characterize speciation of this genus. In addition, a novel clade belonging to *B. cereus* sensu lato was found from various quarters of the ISS surfaces, as well as a virulent *A. fumigatus* from the ISS filter samples which might pose a potential threat to crew health [36]. To consider the potential of whole metagenome data to shed new light on microbial evolution and function, taxonomically binned subsets of metagenomic reads identified by LMAT were examined for the whole genome sequences of *C. ihumii*, *B. cereus* sensu lato, and *A. fumigatus*.

#### *A. fumigatus*

*Aspergillus* was selected for analysis to determine the relationship between detected *Aspergillus* sequence and potentially pathogenic near-neighbor strains, as *A. fumigatus* has been cultured from ISS samples [36]. To improve detection, samples were pooled by location, resulting in approximately 23,000 and 28,000 *Aspergillus* reads in ISS and SAF samples, respectively. Despite the relatively high number of reads corresponding to *Aspergillus*, the total proportion of reads mapped at high quality to each of the four reference genomes was relatively small (less than 5% of ISS reads and approximately 40% of SAF reads). *Aspergillus* reads in the SAF samples consistently covered more bases from the reference genomes than did the ISS reads (~15×); however, in absolute terms, the breadth of coverage was 0.02% or lower and was substantially lower in the pooled ISS sample (Additional file 1: Figure S10). Given this minimal level of coverage, we did not seek to continue a single nucleotide variant-level characterization of the relationship of *Aspergillus* strains within the metagenomic data to potentially pathogenic counterparts.

#### *B. cereus* sensu lato

Sequence reads corresponding to *Bacillus* (Additional file 1: Figure S11A) were studied to determine whether any strain present in these samples could be related to the newly identified *B. cereus* sensu lato [44]. With respect to breadth, reads from either pooled sample covered less than 0.05% of the *B. cereus* sensu lato reference genomes tested. The highest breadth over regions with at least 10× depth for a sample-reference pair was 0.036% for ISS reads mapped to ISSFR-9F (Additional file 1: Figure S11B). The mean breadth of coverage at 10× depth for *B. cereus* sensu lato assemblies ISSFR-23F, ISSFR-25F, ISSFR-3F, and ISSFR-9F was observed to be 0.035% in pooled ISS samples and 0.0026% in pooled SAF samples. The number of variants for *Bacillus*-mapped sequence data was determined with respect to each reference genome, examining fixed or nearly fixed substitutions (Additional file 1: Figure S11C). *B. thuringiensis* YBT1518 and *B. cereus* ATCC 14579 stand out as having a much higher fraction of SNPs, indicating a larger phylogenetic distance from both pooled ISS and pooled SAF samples in this study. No *B. cereus* bases were covered at depth from SAF reads; therefore, no fixed substitutions were detected. *B. cereus* sensu lato and *B. anthracis* exhibit a comparable number of SNPs within samples; however, more reads mapped to the *B. cereus* sensu lato genome with high confidence in the pooled ISS sample. These data suggest the presence of a *Bacillus* variant with the closest relationship to *B. cereus* sensu lato. There is not, however, sufficient evidence to determine whether this strain represents a variant of *B. cereus* sensu lato or a more novel entity.

#### *Corynebacterium* species

*Corynebacterium* was selected for analysis to characterize whether the relatively high number of reads corresponding to this genus could be used to distinguish between *Corynebacterium* species across different sample locations. As reported earlier [14], *Corynebacterium* appear to have been more abundantly represented than *Bacillus* and *Aspergillus* within ISS filter and dust samples, with total LMAT-binned reads numbering in the millions. The SAF-isolated sample, however, was practically devoid of *Corynebacterium* reads (Additional file 1: Figure S12). Only one sample (ISS filter) covered >90% of the *C. ihumii* GD7 reference genome (Additional file 1: Figure S12). The PMA-treated ISS dust sample contained nearly one million LMAT-binned *Corynebacterium* reads. While 97% of these reads mapped at high quality to *C. ihumii* GD7, their spatial distribution was sparse. Contiguously mapped regions were of median length (101 bp), covering only 8110 bases or 0.36% of the reference genome, resulting in islands of extremely deep base coverage (e.g., >600,000× in contig NZ_HG001323.1). ISS dust and PMA-treated ISS filter were the only other samples with >1% coverage of this reference genome (11 and 8%, respectively), precluding further cross-sample comparisons between ISS and SAF environments.

#### *Corynebacterium* allele fractions in ISS samples

**Fig. 6:**
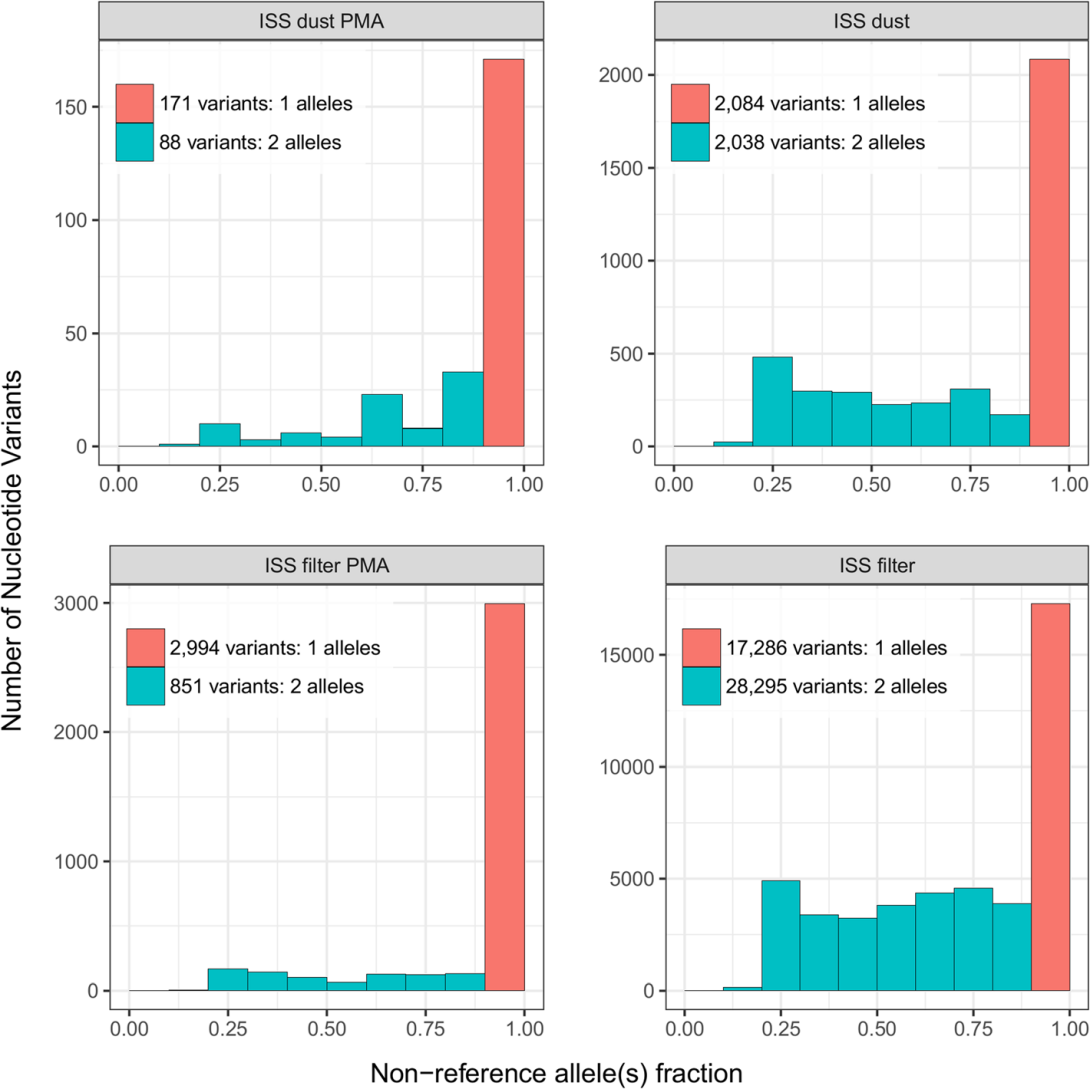
Distribution of *Corynebacterium* non-reference allele abundances across ISS samples, at detected loci in each ISS sample. Sequence reads were mapped to the *C. ihumii* GD7 reference. All alleles meeting depth thresholds at variant positions were identified in each sample. The number of variants at each non-reference allele fraction bin is visualized as stacked histograms where *color* indicates the number of alleles present in the sample at that position; however, all variants in the largest fraction bin (0.9 < non-reference allele fraction ≤ 1.0) were mono-allelic and no variant had more than two present alleles within the sample. The histograms are analogous to non-reference allele frequency spectrums, where allele frequencies are estimated directly from read counts of present alleles

Relevant observations could be made by examining relative *Corynebacterium* allele fractions within ISS samples. We processed variant positions (variants) as called by “freebayes” by decomposing complex variants to their allelic primitives (i.e., gaps and mismatches of length 1) and removing indels. Allele frequencies within samples were then estimated directly as the fraction of reads supporting each observed *Corynebacterium* allele that meets the threshold for presence (Fig. 6, see “Methods”). Within samples, we observed only mono- and bi-allelic variants. The proportion of mono-allelic variants (i.e., 100% of observed reads support a non-reference base call) varied from 38% in the ISS filter to 75% in the PMA-treated ISS filter. In the ISS dust, the proportion was 50%, while in the PMA-treated ISS dust, it was 66%.

**Fig. 7:**
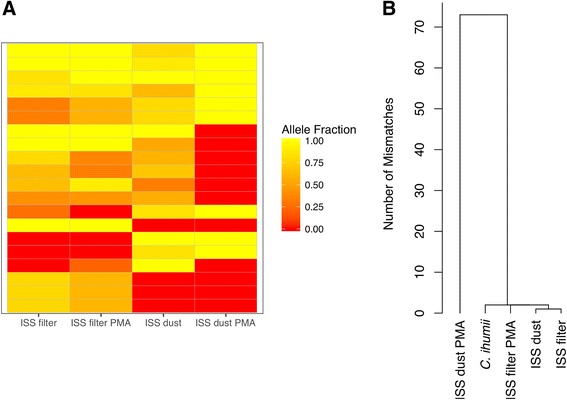
*Corynebacterium* sequence similarity among ISS samples. **a** Top 20 most prevalent non-reference alleles (*rows*) at variant positions present in every ISS sample (*columns*). Alleles are sorted by the number of samples in which each allele is present. *Color* indicates the within-sample relative abundance of reads supporting the allele. **b** Clustering of *C. ihumii* ISS consensus sequences shows the PMA-treated ISS dust sample is distinct. Only positions mapped in all samples where the major allele’s read depth ≤90% were used to calculate Hamming distances

For variants present in regions mapped in the four ISS samples (comprising approximately 5000 bp), we visualized their non-reference alleles sorted by abundance. There were 213 non-reference alleles for 210 variants. Three variants had two non-reference alleles, while the remaining 207 had only one non-reference allele per variant. Of these 213 alleles, only six were observed across the four ISS samples, seven were present in three samples, 24 in two samples, and 176 were unique to each of the ISS samples. Fifty-seven variants in a 16S rRNA gene were identified as having a non-reference allele unique to the PMA-treated ISS dust sample. The allele read depth, i.e., the proportion of reads supporting these unique alleles, ranged from 47 to 100% with median of 74%. The top 20 alleles are shown in Fig. 7a, where clustering of the samples on allele fraction reflects sample location (i.e., dust/debris vs. HEPA filter).

#### *Corynebacterium* GD7 clustering

ISS samples were clustered by mismatch distance (Hamming) of their consensus sequences, using only reference positions that were both (i) mapped in all ISS samples and (ii) near or at fixation (major allele read depth ≥90%) within each sample (Fig. 7b). As opposed to clustering on allele fractions, clustering using fixed and nearly fixed consensus sequences resulted in samples grouping by a lack of PMA treatment first. It should be noted, however, that the bulk of differences in PMA-treated ISS dust fall within a single contig (NZ_HG001324.1), which had an average of 67/72 mismatches compared to the ISS samples and 66/71 compared to the reference *C. ihumii* GD7. Further inspection reveals 20 of these fixed mismatches to be in a 16S rRNA gene (rna56). This relatively high degree of divergence potentially indicates a different strain may be dominant in this sample.

## Discussion

Health of the crew during a space flight mission is of critical importance, both during the mission, as there is limited access to care, and upon return. With increasingly sophisticated molecular tools available to survey-confined built environments such as the ISS, exciting opportunities exist to survey the microbial populations of these environments and their potential impact on human health. Recent studies have begun to explore this built environment, but examination of the microbiome at a species-specific level, in combination with the functional capabilities of those species, has not been studied in depth.

The presented whole metagenome approach, combined with the application of the LMAT platform, allowed for species-specific identification. Further, the use of PMA treatment allowed for the selective detection of DNA sequence associated with viable microorganisms. Both of these factors are critical in determining whether the presence of a microorganism poses a risk to human health. Health-relevant microorganisms detected in PMA-treated ISS samples included a number of *Corynebacterium* species known to cause opportunistic and urinary tract infections, including *C. aurimucosum*, *C. pseudogenitalium*, and *C. urealyticum* [45–47]. Opportunistic *Aspergillus* species were also identified, although at much lower abundance levels. Such species are known to cause invasive infections, though less commonly in immunocompetent individuals [48, 49]. Skin-associated species of *Staphylococcus* were prevalent in PMA-treated ISS dust samples, including *S. aureus*, *S. caprae*, *S. pettenkoferi*, and *S. epidermidis*. These species are commonly associated with human flora and may cause opportunistic infections under certain circumstances [50, 51]. Interestingly, *M. globosa*, the causative agent of dandruff [52], was observed at a high abundance in the ISS dust, again likely due to human contact. Higher incidence of *Malassezia* species associated with the skin of Japanese astronauts was recently reported [53]. The source of *Malassezia* species might be the skin of the crew; however, more in-depth analyses on skin samples of the crew and their inhabitable environments are warranted. In contrast to the human-associated pathogens noted above, *Acinetobacter* were more frequently observed in SAF samples. *Acinetobacter* species are frequent offenders in hospital-acquired infections and are well adapted to propagation in environments subjected to frequent cleaning and disinfection such as cleanrooms and hospitals [54].

Increased levels of human-associated bacteria in the ISS relative to the SAF may reflect constant human contact with ISS spaces, compared to the relatively controlled environment of the SAF cleanroom. Astronauts were required to clean their spacecraft with minimal resources once a week and when necessitated, whereas professional janitorial services were deployed to periodically clean SAF cleanrooms to maintain compliance with their cleanroom certification level. Additionally, increased prevalence of fungal species such as *Rhizophagus irregularis*, *Alternaria arborescens*, *A. pullulans*, and *A. fumigatus* in the SAF may be due to the relative austerity of this environment being more amenable to spore-forming fungi. Increased human-relevant microorganisms in ISS samples might be due to the crew inhabitants, as such observations were not made in the SAF dust. The observed increase in diversity in the SAF sample might be attributed to the exchange of air and soil from the outside environment, which does not occur in the ISS.

The above observations rely on a relative comparison of sequence abundance between microbial species and samples. It is therefore important to note that observed relative abundance levels can be affected by the applied amplification procedures. MDA was used prior to library preparation due to the low biomass limitations and highly precious samples associated with this study. While MDA may certainly impact relative abundance of amplified sequences relative to the original sample, the phi29 polymerase and methods used in this study have been shown to result in the lowest level of amplification bias relative to other available techniques. The potential for bias is similarly true for Nextera DNA library preparation, especially with respect to GC content bias [55], which also includes a degree of sample amplification prior to sequencing. It is therefore important to note that, for the distinctions in relative abundance called out in this study, the actual quantity of precise fold change in abundance may differ somewhat from what was observed via the sequence data.

It should further be noted that selection by PMA treatment may not be uniform across every family of microbes. Spore-forming microorganisms, for instance, will react differentially to PMA exposure, as PMA may not sufficiently penetrate non-viable but intact spores [20]. Archaea are also indicated as demonstrating a distinct profile, though such organisms are anticipated to be extremely low abundant in these samples [56]. While PMA treatment may impact observed abundance distinctions to a degree, this method creates a unique opportunity to enrich for viable microorganisms in a culture-free context and thus carries a value despite possible impact on capacity for quantitative assessment.

The detected fungi are not typically associated with human disease, though *Alternaria* are capable of causing invasive alternariosis [57] and *A. pullulans* may cause complicating infections in patients undergoing chemotherapeutic regimens [58]. *Aspergillus*, particularly the identified and previously cultured *A. fumigatus*, are well known to exhibit virulence and cause disease in humans [59]. Additional fungal content within ISS samples included the potential food spoilage agent *Penicillium* [60], a notable observation as food spoilage is a relevant concern within the ISS. Overall, observation of sequence data mapping to the bacterial and fungal species above was in agreement with previous 16S rRNA iTag sequencing results [14], indicating consistency of the whole metagenome data.

Although the majority of microbial species detected in this study cause mainly opportunistic infections, this does not mean their presence should be discounted with respect to astronaut health. It is known that microgravity, radiation, restricted diet, and limited hygiene practices can impact the immune systems of otherwise healthy individuals, altering cytokine and chemokine expression [12, 18, 61]. Microorganisms associated with opportunistic infections have been previously observed in the ISS [36, 62], and their presence could be a concern dependent on immune status of the exposed individual.

A factor critical to assessing risk of a given microbial population is evaluation of its genetic content, particularly with respect to antimicrobial resistance. This information cannot be obtained using 16S rRNA or ITS sequencing nor by predictive metabolic profiling [63] as shown recently [13]. Important steps have been taken recently toward evaluating virulence factors in assembly facilities of terrestrial cleanrooms, finding that virulence components from a range of human pathogens exist in these spaces [17]. Screening of our PMA-treated whole metagenome data for AMR revealed increased prevalence of genes encoding resistance factors in the ISS, particularly in dust samples, while the PMA-treated dust sample from the SAF contained few AMR-related genes. One possible explanation is that AMR factors may be more common in microbial populations with human contact, as they will encounter selective pressure from medical, environmental, and other treatments [64]. This has been demonstrated in previous studies of indoor environments, where human-introduced antimicrobial chemicals result in the elevated presence of AMR-associated genes [65]. Though human traffic is more frequent and diverse in the SAF, it is also better controlled on Earth relative to the ISS. PMA treatment resulted in a reduction in detected AMR categories in the ISS filter, possibly indicating that while the total historical population exhibited AMR potential, the currently viable population contained fewer such signatures. This may be due to the reduced bacterial fitness associated with maintaining resistance in the absence of selective pressure [66, 67].

It was observed that functional gene categories detected in the ISS dust were more likely to be associated with active growth and metabolism. This may be due to proximity of these samples with microbiomes of crew members, which may be better adapted to a metabolically robust life cycle. Importantly, functional genomic observations were made in PMA-treated samples, such that detected genes are likely derived from viable microorganisms and might impact human occupants.

Among the AMR gene categories uniquely identified by LMAT after screening against the CARD and VFDB, relatively higher numbers of reads were assigned to genes (>100 reads) related to the ATP-binding cassette superfamily (ABCs), multidrug and toxic compound extrusion (MATE) family, rRNA methyltransferase, methionine sulfoxide reductase (*msr*A), fluoroquinolone resistance (*pat*A and *pat*B), and clindamycin resistance (*erm*ABC) in the ISS dust (Fig. 5a). The ABCs genes, found in both ISS samples, have been reported to regulate the access of drugs to microorganisms [68]. The MATE mutant strains in certain microorganisms exhibited increased sensitivity to the toxic organic cations acriflavine and methyl viologen, but not fluoroquinolones, tetracycline, berberine, or sodium deoxycholate [69]. The rRNA methyltransferases (MTases), a large protein superfamily, commonly use *S*-adenosyl-l-methionine (SAM) as the methyl group donor. The SAM-dependent MTases methylate both nucleic acids (DNA, RNA) and proteins and thus modulate their activity, function, and folding. As shown in Fig. 4, DNA and RNA metabolic microbial pathways were in higher abundance in ISS dust samples, which also exhibited more detected AMR gene categories. Methylation of nucleotides of 16S rRNA in aminoglycoside-producing microorganisms confers resistance to their own toxic product(s) [70]. The methionine sulfoxide reductase gene (*msr*A) is involved in the oxidation of sulfur-containing residues and their regulation has emerged as a key mechanism of redox control [71]. Methionine oxidation is a form of oxidative damage of proteins, a modification that alters protein structure or function, a tool in redox signaling, and a mechanism that controls protein function [71]. Overexpression of the ABC transporter genes *pat*A and *pat*B confers efflux-mediated fluoroquinolone resistance in *Streptococcus* species and is also linked to stress responses and multidrug resistance [72]. Isolation of *Streptococcus* was also reported from the ISS environmental samples [14]. In a recent study [73], *S. aureus* isolates were examined for inducible clindamycin resistance and the presence of erythromycin ribosome methylase (*erm*ABC) genes. Isolation of *S. aureus* from ISS dust samples and not from SAF samples was reported in this study, and the retrieval of clindamycin resistance genes from the ISS dust is in accordance with this observation.

Other genes uniquely identified by LMAT were related to transport protein *qac*B, multidrug efflux protein, lincosamide, and beta-lactamase resistance (Fig. 5b). The quaternary ammonium compound resistance gene (*qac*) also codes for resistance to a broad spectrum of other cationic compounds such as intercalating dyes, diamidines, and biguanides [74]. In *Staphylococcus* species, several plasmid-encoded Qac efflux pumps have been described, belonging to two major protein families (QacA and QacB). The ISS utilizes mainly benzalkonium chloride wipes, a Qac compound as cleaning agents; hence, the presence of the *qac* genes is not surprising. The multidrug efflux systems play a major role in resistance to a wide range of noxious compounds in several Gram-negative species. It has been reported that the drug resistance and virulence phenotypes of *Salmonella* mutants defective either in resistance-nodulation-division (RND)-type systems or in drug efflux systems belonging to the major facilitator, multidrug and toxic compound extrusion (MATE), and ATP-binding cassette (ABC) superfamilies [75]. Lincosamide resistance was reported to harbor *lsa*C and *tet*W genes in *Streptococcus* species [76]. Antibiotics and antibiotic-resistant bacteria might enter into the ISS via various sources (cargo, human occupants, etc.), where resistance genes can potentially spread and exchange between microbes. These include but are not limited to the sulfonamide resistance genes (*sul*1 and *sul*2), tetracycline resistance genes (*tet*M and *tet*C), and resistance genes for extended spectrum beta-lactams (*bla*_oxa-58_, *bla*_shv-34_, and *bla*_ctx-m-32_). Furthermore, the presence of these genes in the cultivable population of ISS samples should be explored to confirm the presence of these genes via metagenome analysis.

As was previously noted, many of the virulence-associated genes identified in this study play a role in conferring AMR. A subset of these genes may also contribute to a virulence phenotype independent of resistance. Adenine methylase, for instance, has been shown to impact the expression of numerous genes regulating cellular activities relevant to virulence [77], including cell invasion and protein secretion in *Salmonella typhimurium* [78]. Bacterial elastases, also detected in the current study, have similarly been implicated in cellular invasion by *Pseudomonas aeruginosa* [79] through proteolytic activity at the site of infection [80]. A variety of genes encoding virulence-associated outer membrane proteins, such as genes encoding Lom-like proteins detected here, may enhance virulence by improving survival within host macrophages [81]. rRNA methylases have been broadly implicated in virulence phenotypes across many bacterial species, including *S. aureus* [82], in addition to conferring resistance to aminoglycosides [83]. Expression of bacterial transposases may also broadly impact virulence through regulating gene expression, allowing for inter-bacterial transfer of mobile virulence elements and promoting in vivo adaptation [84]. Detection of the virulence-associated genes described here is highly relevant in the context of confined human habitation within the ISS, as these components will impact the ability of microorganisms such as *Pseudomonas* and *Staphylococcus* to effect particularly problematic and difficult-to-treat clinical manifestations in the crew through host cell invasion or other mechanisms. These factors should, therefore, be taken into account, though it should also be noted that such genes may also play general roles in other bacterial housekeeping functions under normal metabolic conditions.

The availability of gene content is a valuable resource for interpreting the functional capabilities of microorganisms in confined built environments. A challenge in interrogating these data is that assigning gene-level calls within whole metagenome data is inherently difficult given the depth required for high confidence assignment. Further efforts to identify the most salient functional gene categories, and application of targeted sequencing efforts toward characterizing these genes at great depth, may yield additional insight into the evolution and adaptation of microbial populations in such spaces. Ongoing studies are underway to assess space station and crew microbiomes over time during travel to and residence within the station, which may establish further connections between human health and function and dynamics of the microbial population that surrounds the human host in the ISS. Given the known impact of space travel and residence on immune function, this represents a critical piece of information and is the subject of great interest for future exploration.

The availability of sequence data corresponding to *Bacillus* and *Corynebacterium* across multiple samples raised the possibility that strain-level comparisons might be made across different locations. However, there was no coincident coverage corresponding to the assessed reference genomes across samples, making it difficult to identify sample-specific mutations. For example, at the genus level, LMAT classified approximately 25,000 and 71,000 reads as *Bacillus* in the pooled ISS and pooled SAF samples, respectively. These reads were mapped to *Bacillus* reference genomes through short read alignment. Despite the nearly three times as many SAF reads compared to ISS reads, a smaller proportion of SAF reads were mapped to *Bacillus* genomes at high quality. With respect to read counts, fewer SAF reads mapped at high quality than ISS reads. However, relaxing the mapping quality criteria reverses this relationship (Additional file 1: Figure S11A). These results are congruent with taxonomic binning with LMAT, where it was found that the majority of *Bacillus* reads associate with *Bacillus ginsengihumi*. As reported previously, *B. ginsengihumi* was isolated from ISS samples by standard culture techniques [14].

Since *B. cereus* sensu lato was previously isolated from the ISS [85], effort was taken to determine whether it could be observed in the current dataset. Our short-read alignment mapping of *Bacillus* reads in ISS samples supports classification by LMAT of *B. cereus* sensu lato at the species/strain level. Although few reference genomes were used in the alignment mapping, compared to the comprehensive LMAT database, a higher percentage of reads aligned to *B. cereus* sensu lato strains than non-*B. cereus* sensu lato strains. Of the bases mapped at high quality and depth, more variants were identified when using non-*B. cereus* sensu lato strains as references. These data point toward a variant most closely related to *B. cereus* sensu lato; however, sequencing at larger library sizes or higher depth to increase the probability of broader coverages would be required to assign taxonomy with greater confidence.

Breadth of reference coverage for *Corynebacterium* was uneven across samples. However, *Corynebacterium* was sufficiently abundant on ISS to track a 5000-bp region common to ISS samples. It was possible to cluster samples via the Hamming distance between consensus sequences of fixed and nearly fixed positions and on non-reference allele frequencies for variant positions common to ISS samples, including those not near fixation. Samples clustered according to location when examining individual alleles, and according to PMA treatment status when comparing consensus sequences of fixed sites. These results suggest that fixed substitutions may separate viable samples from those that include non-viable sequences, while shared unfixed mutations, possibly from low-abundance competing strains, may separate the ISS filter from ISS dust environments. Future studies that more broadly cover the reference genome or pan-genome could confirm whether this relationship holds true.

The observed breadth of coverage for the species examined in this study may seem relatively low when compared to the absolute quantity of sequence data available. This was due in part to our application of a high threshold for depth of coverage (10×), which is important for declaring high-confidence base calls but filters out large portions of shallow coverage sequence. For *Aspergillus* and *Bacillus*, on average, 36% of mapped reads passed the Snippy map quality threshold (mapping score ≥60) and 6% of high-quality bases passed the Snippy read depth threshold (≥10 reads). Additionally, in several cases, particularly for *Bacillus*, the strain inferred from metagenomic sequence data was sufficiently divergent from reference strains as to preclude mapping to a reference with high quality. If future studies are undertaken with the explicit goal of making strain-level comparisons, for example with MIDAS [40], panphlan [86], or similar suites, consideration should be taken toward extensively over-sequencing such samples so as to increase the likelihood, not only for high coverage within a sample but also for high breadth of shared coverage across samples. This may not, however, improve reference coverage in the event that a highly divergent novel strain is under study.

## Conclusion

Metagenomic analysis of a controlled environment such as the ISS allows us to study the microbial composition of a unique circumstance where human habitation occurs during space travel. A whole metagenome approach permits for high taxonomic resolution and the ability to monitor changes in functional characteristics of microorganisms, which is not possible with amplicon sequencing. A comprehensive picture is necessary to guide less expensive, but focused assays. Such information will be crucial while planning for long-term exploration. Comprehensive sterilization is neither a viable nor desirable solution for such an endeavor; thus, it is critical to understand human co-inhabitance with the surrounding microbial community. Studies such as these may inform future approaches toward reducing the relative presence of pathogenic microbes and further understanding which microbiome compositions are amenable to healthy conditions for future space travelers. This is the first study to analyze antibiotic resistance and virulence genes from ISS whole metagenome sequence data. These data are important to assessment of the pathogenic potential of space habitats and may shed light on the use of countermeasures during future long-term space missions.

## Additional file

Additional file 1:
**Figure S1**. Sequence reads obtained from ISS and SAF samples were uniquely mapped to microorganisms at species-level resolution using the LMAT platform. **Figure S2**. Comparison of the microbial profile represented by the top 100 microbial species observed in each total and viable (PMA-treated) sample. **Figure S3**. Taxonomic network map showing common taxa between PMA-treated samples. Sequence data were aligned to reference sequence using DIAMOND, and class-level nodes visualized in a network plot. **Figure S4**. Genus level distribution of sequence data for PMA-treated ISS filter sample, as determined by DIAMOND mapping for taxonomic network analysis. **Figure S5**. Genus level distribution of sequence data for PMA-treated ISS dust sample, as determined by DIAMOND mapping for taxonomic network analysis. **Figure S6**. Genus level distribution of sequence data for PMA-treated SAF dust sample, as determined by DIAMOND mapping for taxonomic network analysis. **Figure S7**. Simplified variant analysis workflow. For each sample, metagenomic reads extracted from LMAT are aligned to a putative representative reference genome and variants are identified using Snippy [33] [Seemann T: snippy: Rapid bacterial SNP calling and core genome alignments; 2015: https://github.com/tseemann/snippy/releases/tag/v3.1.] (See Methods). **Figure S8**. Antimicrobial resistance gene categories detected in both total and viable (PMA-treated) ISS and SAF samples. Microbial genes uniquely identified by LMAT above the specified read match threshold were screened against the Comprehensive Antimicrobial Resistance Database (CARD). **Figure S9**. Virulence gene categories detected in both total and viable (PMA-treated) ISS and SAF samples. **Figure S10**. Sequence reads mapped to *Aspergillus* by LMAT were aligned to *Aspergillus* reference genomes, shown along the horizontal axis. **Figure S11A**. Sequence reads mapped to *Bacillus* by LMAT were aligned to *Bacillus* reference genomes, shown along the horizontal axis. **Figure S11B**. Sequence reads mapped to *Bacillus* by LMAT were aligned to *Bacillus* reference genomes, shown along the horizontal axis. **Figure S11C**. Sequence reads mapped to *Bacillus* by LMAT were aligned to *Bacillus* reference genomes, shown along the horizontal axis. **Figure S12**. Sequence reads mapped by LMAT to *Corynebacterium* were identified. (DOCX 3758 kb)
