# Erratum to: Whole metagenome profiles of particulates collected from the International Space Station

**DOI:** 10.1186/s40168-017-0330-2

**Published:** 2017-09-01

**Authors:** Nicholas A. Be, Aram Avila-Herrera, Jonathan E. Allen, Nitin Singh, Aleksandra Checinska Sielaff, Crystal Jaing, Kasthuri Venkateswaran

**Affiliations:** 1 Physical and Life Sciences Directorate, Lawrence Livermore NationalLaboratory, Livermore, CA USA; 2 Computation Directorate, Lawrence Livermore National Laboratory, Livermore, CA USA; 3 Biotechnology and Planetary Protection Group, Jet Propulsion Laboratory, California Institute of Technology, M/S 89-2, 4800 Oak Grove Dr, Pasadena, CA 91109 USA; 4 Present Address: Department of Ecology, Evolution and Organismal Biology, Iowa State University, Ames, IA USA

## Erratum

Following publication of the original article [1], the authors reported that the X-axis label was missing from Fig. 4. The new Fig. 4 is attached.

**Fig. 4:**
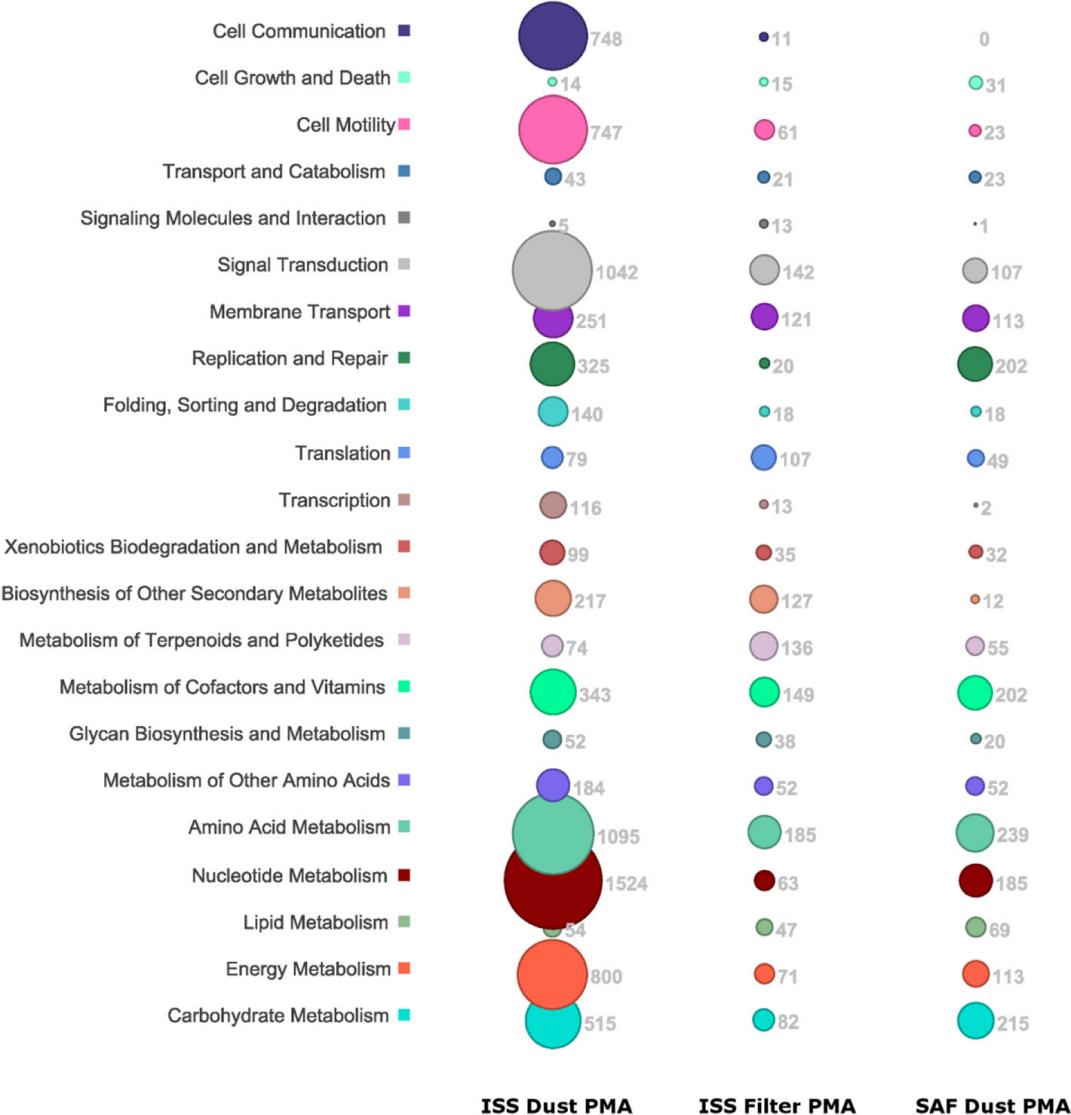
Microbial gene pathways observed in the whole metagenomes of ISS and SAF samples. Reads matching microbial gene targets above an identity threshold of 0.9 were assigned to KEGG orthologies. KO number was used to assign a gene function category, shown along the *vertical axis*. Read abundance is graphically represented on a square-root scale. Absolute read counts are shown adjacent to each corresponding *circle*
