# Supplementary material for: Whole metagenome profiles of particulates collected from the International Space Station
Source: Microbiome. 2017 Jul 17;5:81. doi: 10.1186/s40168-017-0292-4 (PMC5514531; doi:10.1186/s40168-017-0292-4)
Supplement: Additional file 1: — Figure S1. Sequence reads obtained from ISS and SAF samples were uniquely mapped to microorganisms at species-level resolution using the LMAT platform. Figure S2. Comparison of the microbial profile represented by the top 100 microbial species observed in each total and viable (PMA-treated) sample. Figure S3. Taxonomic network map showing common taxa between PMA-treated samples. Sequence data were aligned to reference sequence using DIAMOND, and class-level nodes visualized in a network plot. Figure S4. Genus level distribution of sequence data for PMA-treated ISS filter sample, as determined by DIAMOND mapping for taxonomic network analysis. Figure S5. Genus level distribution of sequence data for PMA-treated ISS dust sample, as determined by DIAMOND mapping for taxonomic network analysis. Figure S6. Genus level distribution of sequence data for PMA-treated SAF dust sample, as determined by DIAMOND mapping for taxonomic network analysis. Figure S7. Simplified variant analysis workflow. For each sample, metagenomic reads extracted from LMAT are aligned to a putative representative reference genome and variants are identified using Snippy [33] [Seemann T: snippy: Rapid bacterial SNP calling and core genome alignments; 2015: https://github.com/tseemann/snippy/releases/tag/v3.1.] (See Methods). Figure S8. Antimicrobial resistance gene categories detected in both total and viable (PMA-treated) ISS and SAF samples. Microbial genes uniquely identified by LMAT above the specified read match threshold were screened against the Comprehensive Antimicrobial Resistance Database (CARD). Figure S9. Virulence gene categories detected in both total and viable (PMA-treated) ISS and SAF samples. Figure S10. Sequence reads mapped to Aspergillus by LMAT were aligned to Aspergillus reference genomes, shown along the horizontal axis. Figure S11A. Sequence reads mapped to Bacillus by LMAT were aligned to Bacillus reference genomes, shown along the horizontal axis. Figure S11B. Sequence re [file 40168_2017_292_MOESM1_ESM.docx]

**Figure S1.** Sequence reads obtained from ISS and SAF samples were uniquely mapped to microorganisms at species-level resolution using the LMAT platform. Relative abundance of the top 100 microbial species observed in total and viable (PMA-treated) populations was determined. Detected organisms were then binned at the genus level and the proportion of total mapped reads attributed to each of these genera was plotted. Relative read abundance is shown along the vertical axis and individual samples along the horizontal axis.


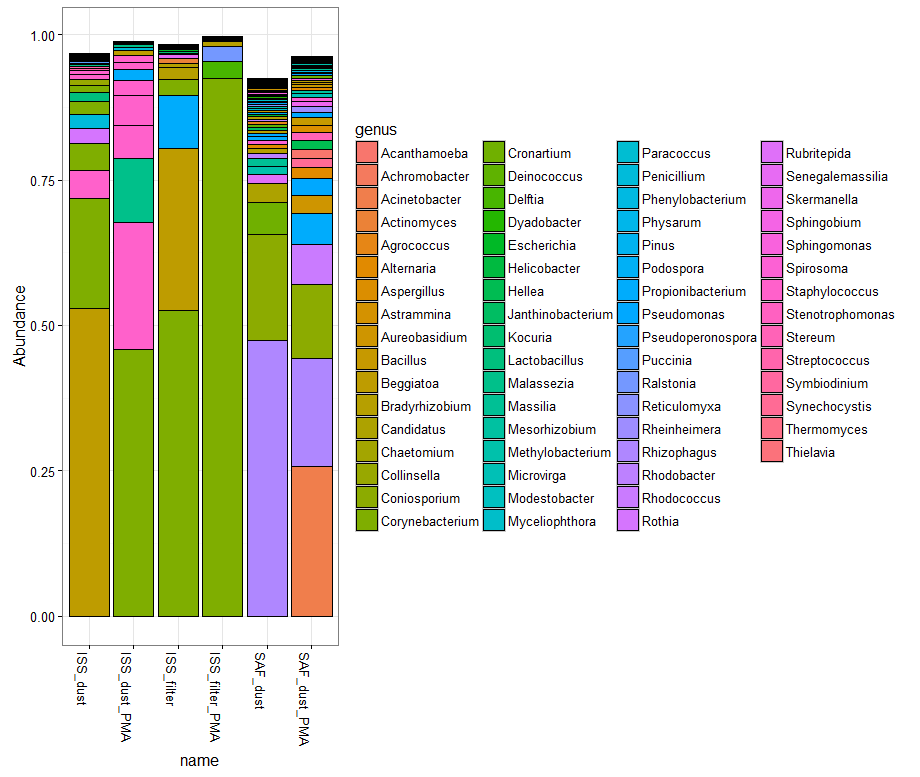


**Figure S2.** Comparison of the microbial profile represented by the top 100 microbial species observed in each total and viable (PMA-treated) sample. All detected organisms were binned at the genus level. Each panel represents one independent sample. The top detected species across all samples are shown on the horizontal axis and relative read abundance along the vertical axis.

**Figure S3.** Taxonomic network map showing common taxa between PMA-treated samples. Sequence data were aligned to reference sequence using DIAMOND, and class-level nodes visualized in a network plot. PMA-treated ISS dust (blue), ISS filter (green) and SAF dust (red) samples are shown with edges drawn to nodes representing taxonomic classes common between samples (grey). Classes unique to a given sample are shown as nodes with colors corresponding to the respective sample.

**
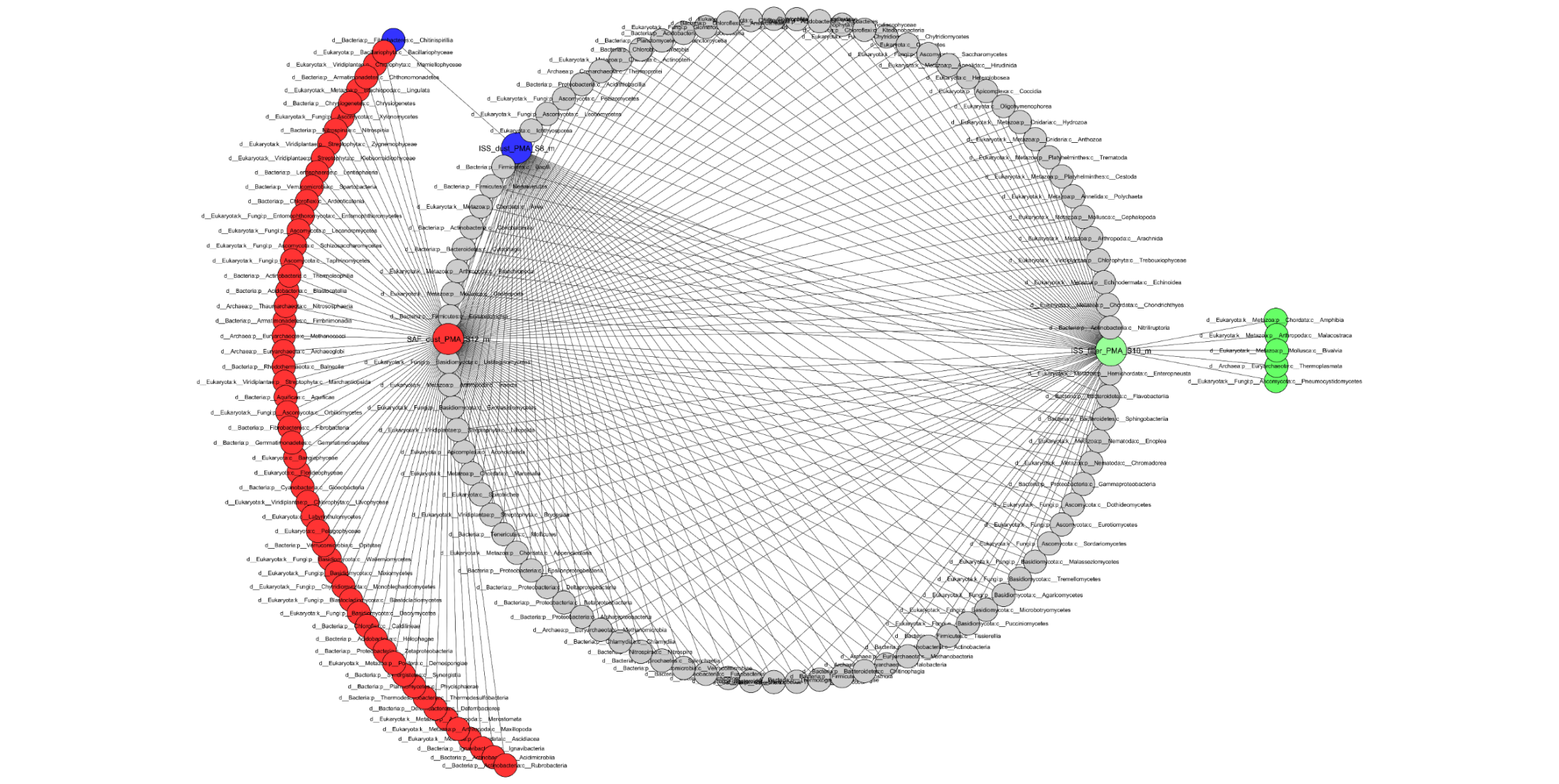
**

**Figure S4.** Genus level distribution of sequence data for PMA-treated ISS filter sample, as determined by DIAMOND mapping for taxonomic network analysis. Observation of *Plasmodium* sequence data determined as likely due to human sequence contamination within the *Plasmodium* reference genome, as a sequence-contamination pre-filtering step in orthogonal LMAT mapping analysis revealed minimal *Plasmodium* detection events. Observed primate sequence data is also likely due to alignment of human metagenomic data.

**
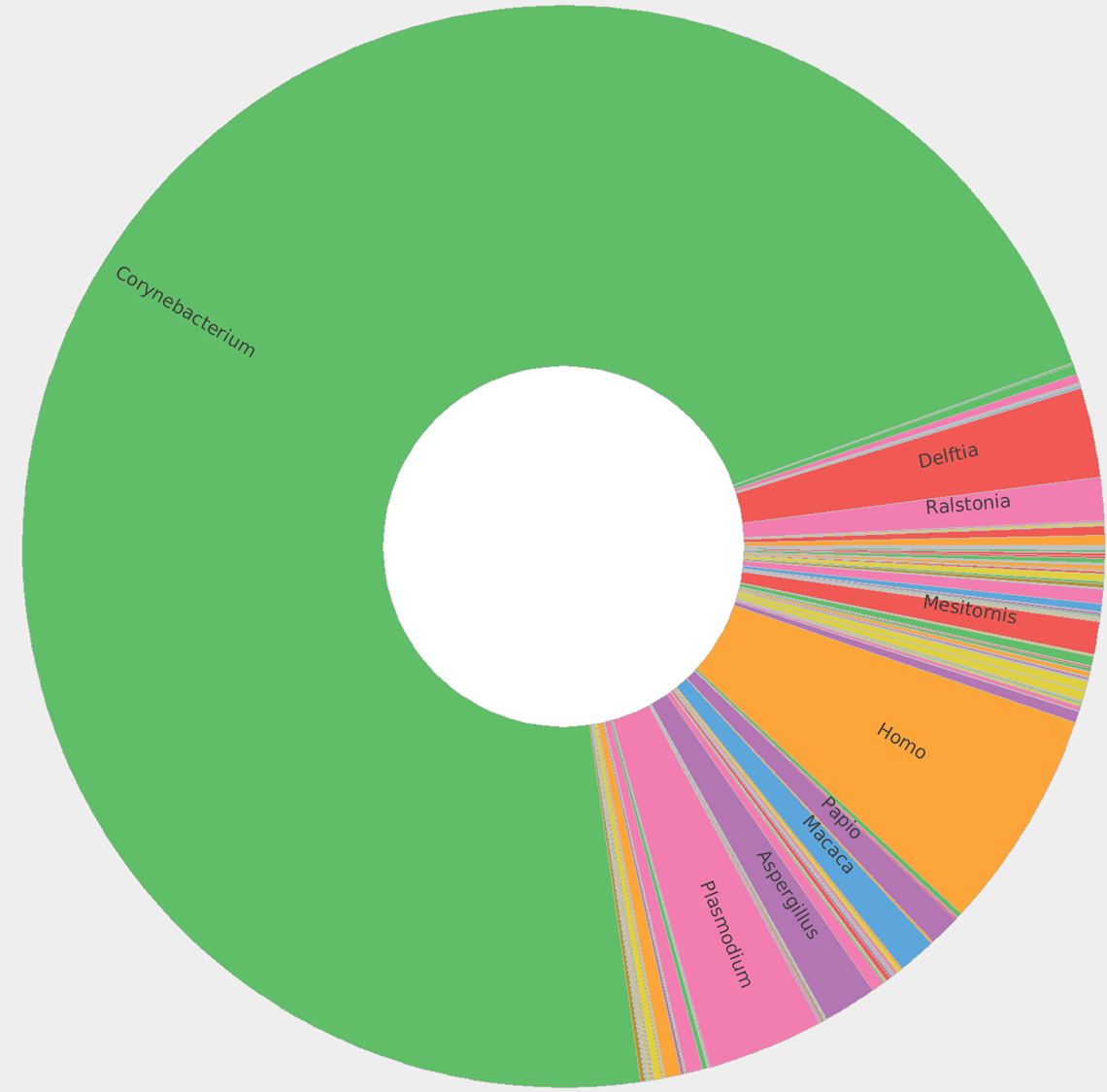
**

**Figure S5.** Genus level distribution of sequence data for PMA-treated ISS dust sample, as determined by DIAMOND mapping for taxonomic network analysis.


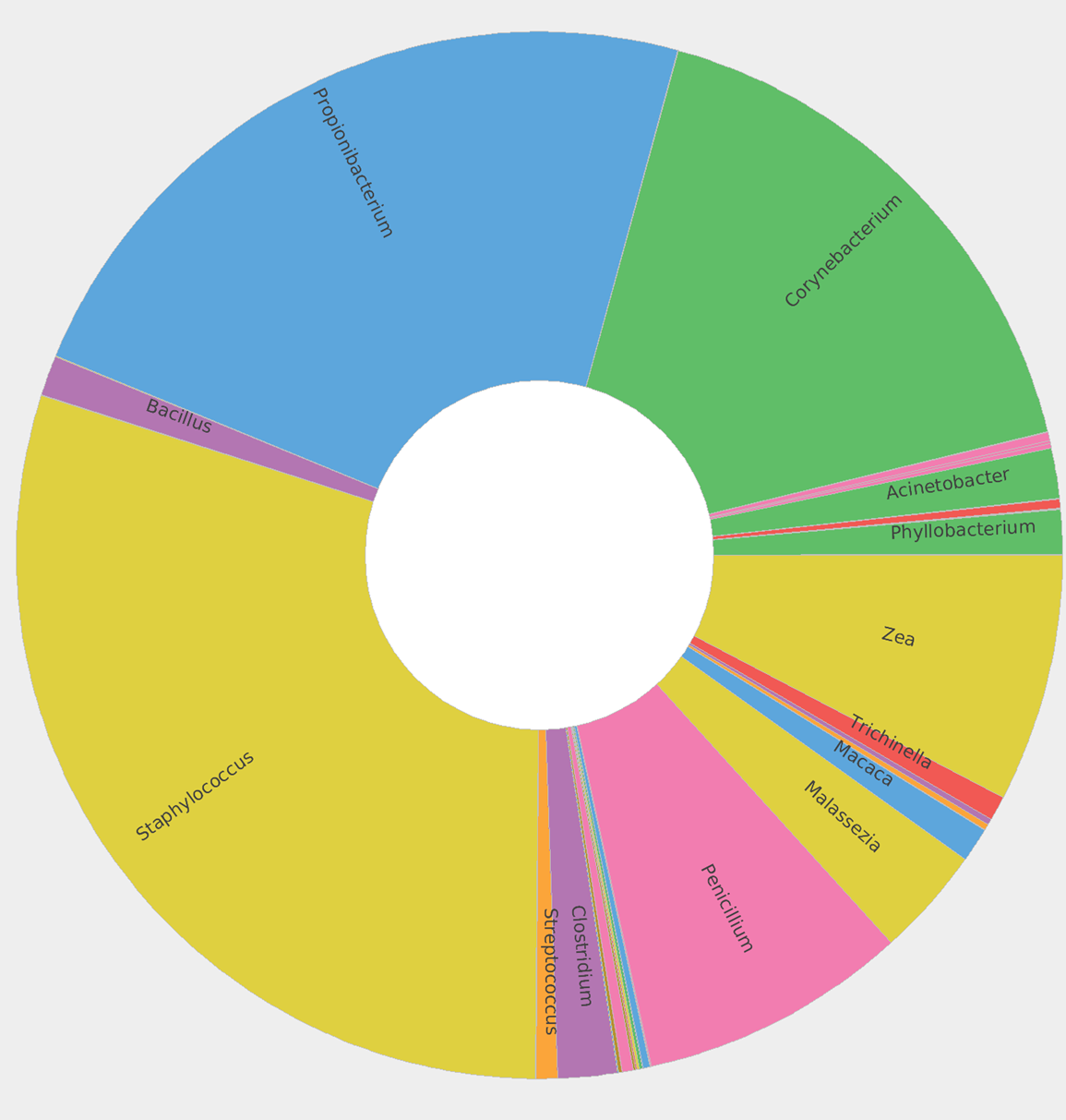


**Figure S6.** Genus level distribution of sequence data for PMA-treated SAF dust sample, as determined by DIAMOND mapping for taxonomic network analysis.


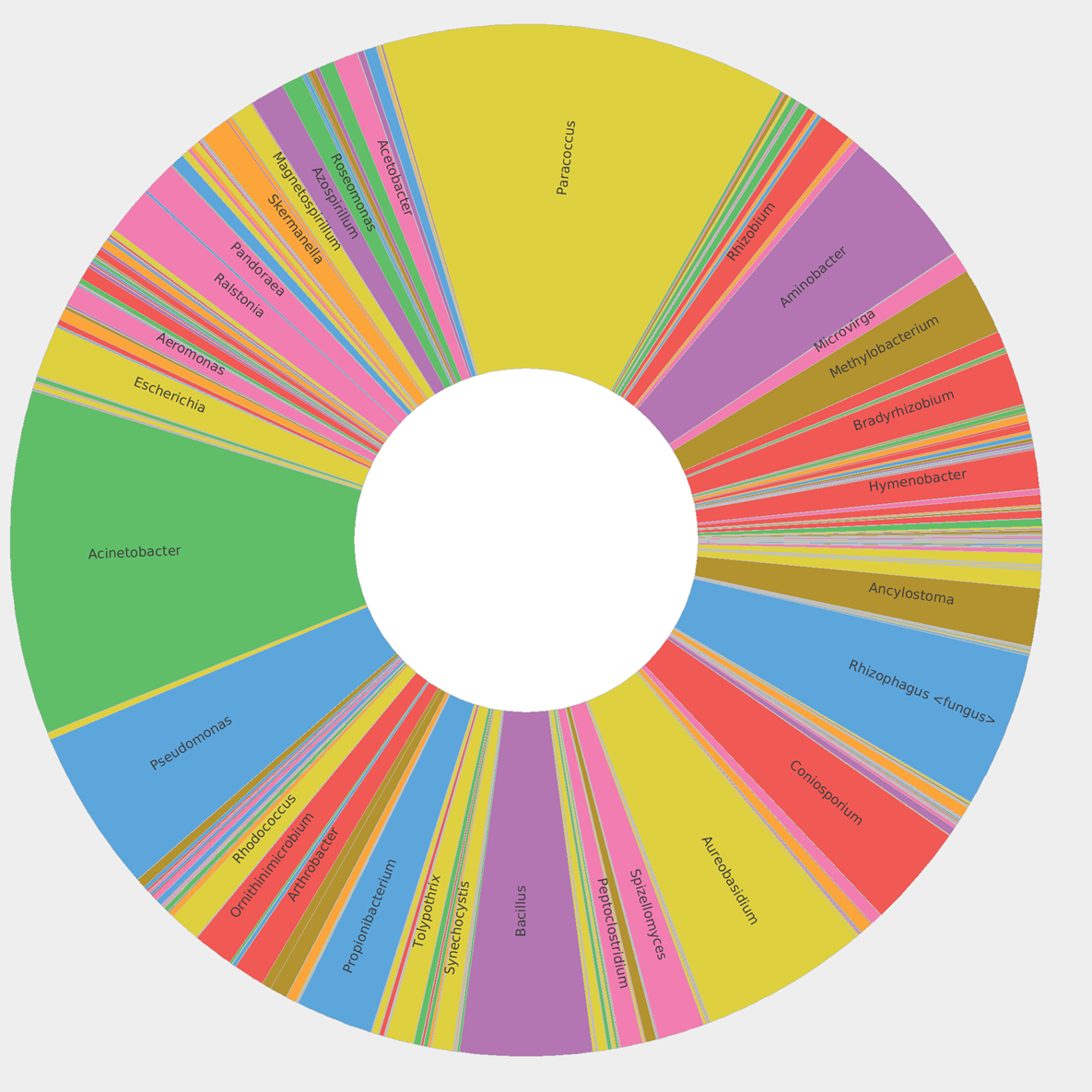


**Figure S7.** Simplified variant analysis workflow. For each sample, metagenomic reads extracted from LMAT are aligned to a putative representative reference genome and variants are identified using Snippy [33] [Seemann T: snippy: Rapid bacterial SNP calling and core genome alignments; 2015: <https://github.com/tseemann/snippy/releases/tag/v3.1>.] (See Methods). Read and base mappings are filtered on map quality and depth. Variants are filtered and simplified to include only single nucleotide substitutions. (a) Mapped regions are used to calculate gene coverage, fixed positions, and to find regions mapped in other samples. (b) The sample consensus sequence only includes positions that were mapped at high quality, fixed in the population, and ambiguities resolved by choosing the allele at fixation (See Materials and Methods).

**
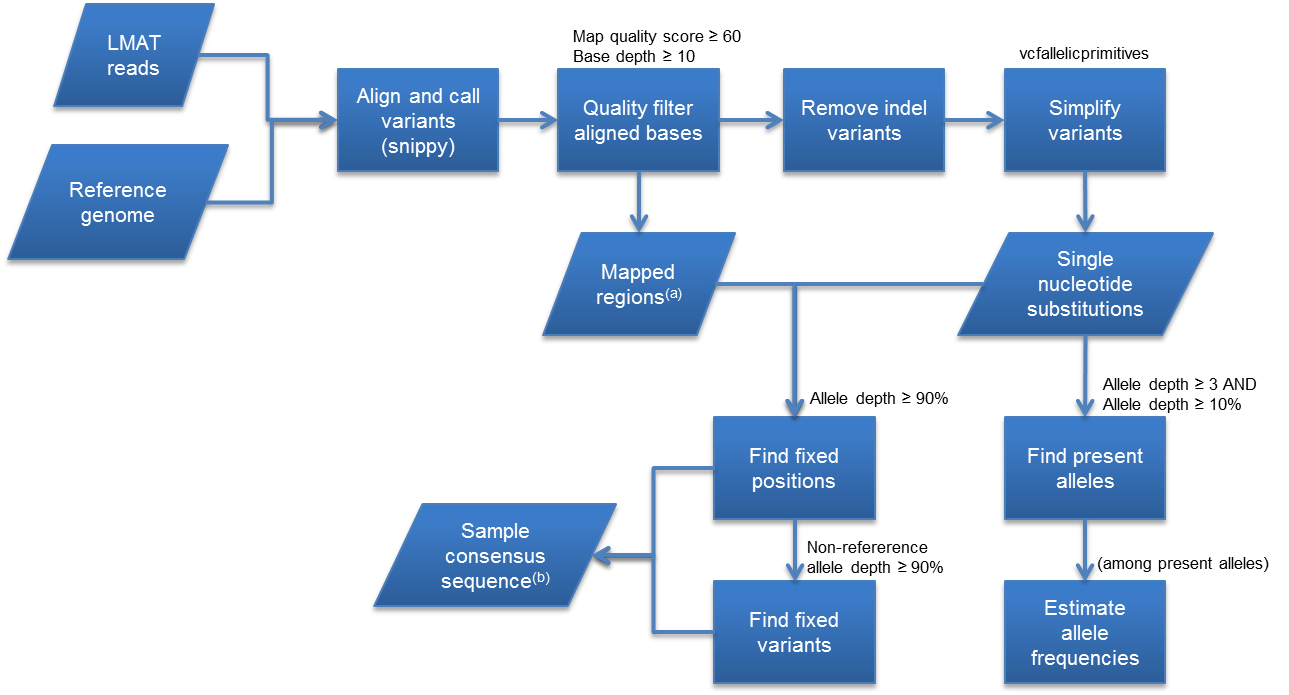
**

**Figure S8.** Antimicrobial resistance gene categories detected in both total and viable (PMA-treated) ISS and SAF samples. Microbial genes uniquely identified by LMAT above the specified read match threshold were screened against the Comprehensive Antimicrobial Resistance Database (CARD). Detected genes were binned into functional categories. Absolute read counts corresponding to each functional AMR category are shown. The color scales indicate absolute read abundance. Gene categories are shown along the vertical axis in alphabetical order and PMA-treated samples along the horizontal axis grouped by sample.


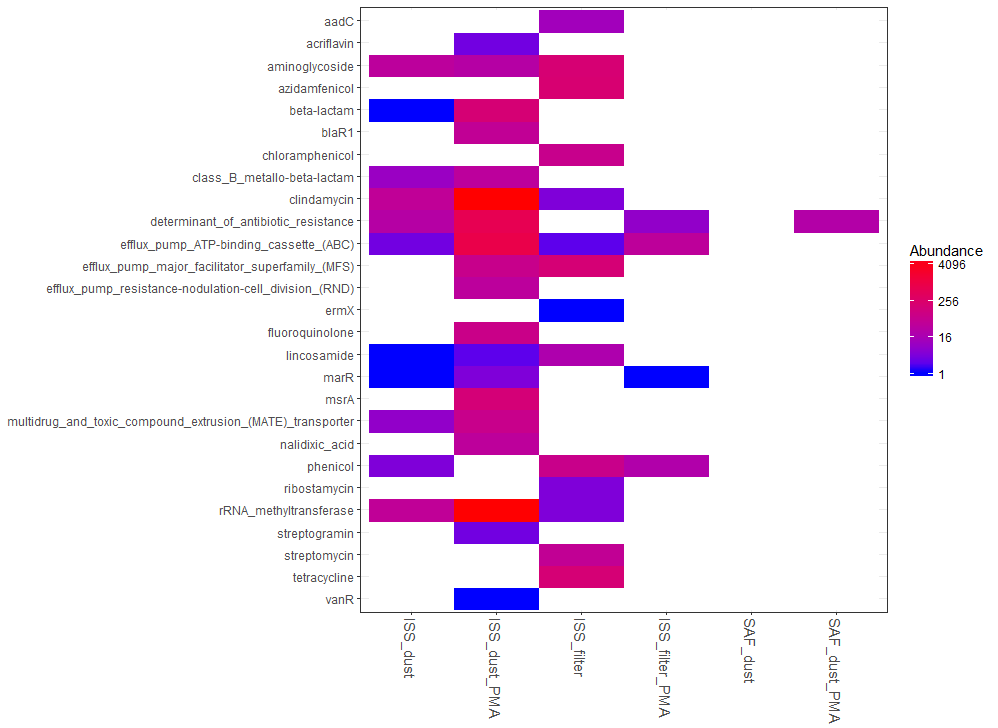


**Figure S9.** Virulence gene categories detected in both total and viable (PMA-treated) ISS and SAF samples. Genes uniquely identified by LMAT above the specified read match threshold were screened against the Virulence Factor Database (VFDB). Detected genes were binned into functional categories. Absolute read counts corresponding to each functional AMR category are shown. The color scales indicate absolute read abundance. Gene categories are shown along the vertical axis in alphabetical order and PMA-treated samples along the horizontal axis grouped by sample.


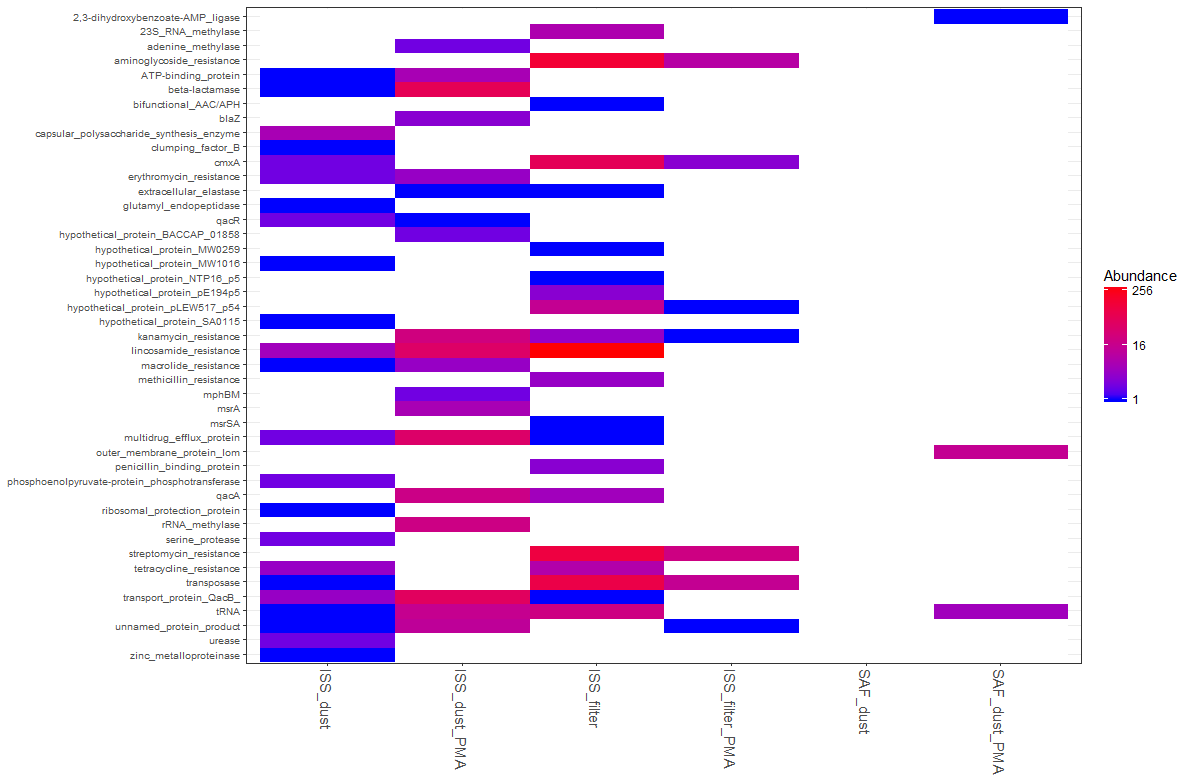


**Figure S10.** Sequence reads mapped to *Aspergillus* by LMAT were aligned to *Aspergillus* reference genomes, shown along the horizontal axis. All ISS samples were pooled and all SAF samples were pooled for analysis. The percent of each reference genome covered by all aligned bases, and bases with read depth ≥10x (High Quality) from ISS and SAF sequence datasets is shown. *A. fumigatus* strains IF1SWF4 and ISSFT021 were isolated on the space station.

**
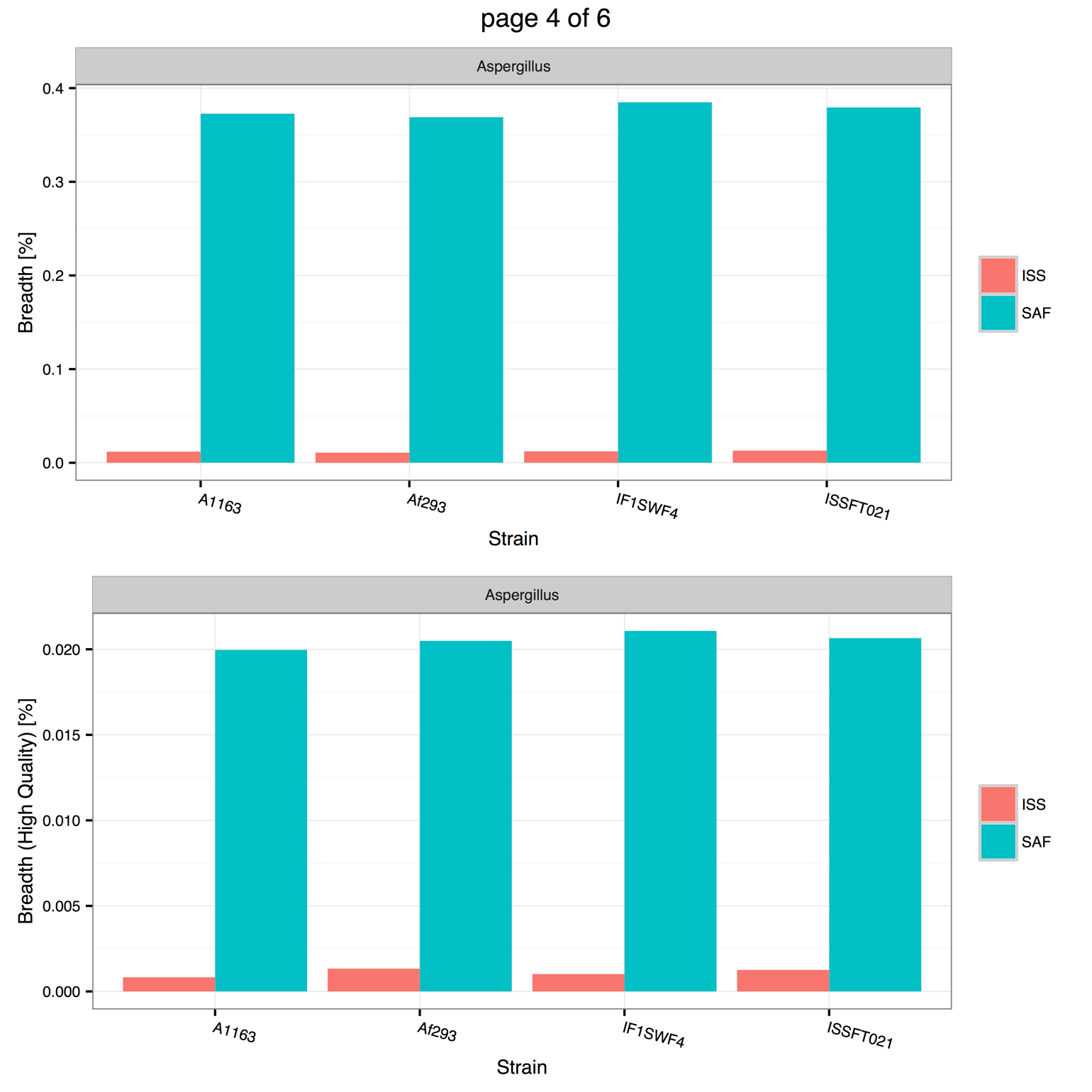
**

**Figure S11A.** Sequence reads mapped to *Bacillus* by LMAT were aligned to *Bacillus* reference genomes, shown along the horizontal axis. All ISS samples were pooled and all SAF samples were pooled for analysis. The total quantity (left panels) and percent (right panels) of all reads aligned to specified reference genomes at any map quality and at a map quality score ≥60. Strains with names beginning with ISSFR were isolated from the space station.

**Figure S11B.** Sequence reads mapped to *Bacillus* by LMAT were aligned to *Bacillus* reference genomes, shown along the horizontal axis. All ISS samples were pooled and all SAF samples were pooled for analysis. The percent of each reference genome covered by all aligned bases, and bases with read depth ≥10x (High Quality) from ISS and SAF sequence datasets are shown. Strains with names beginning with ISSFR were isolated from the space station.

**Figure S11C.** Sequence reads mapped to *Bacillus* by LMAT were aligned to *Bacillus* reference genomes, shown along the horizontal axis. All ISS samples were pooled and all SAF samples were pooled for analysis. Variant positions were identified for the reference regions mapped at high quality and base depth. The number of fixed substitutions at these positions is shown (major allele constitutes 90% or more of the read depth).

**Figure S12.** Sequence reads mapped by LMAT to *Corynebacterium* were identified. **Top:** Total number of reads mapped by LMAT are shown for each sample. **Bottom:** Reads mapped to *Corynebacterium* by LMAT were aligned to the *C. ihumii* GD7 reference to determine proportion of the reference sequence covered by these data. The percent of reference bases covered by sequence data from each sample is shown.
